# Imaging single CaMKII holoenzymes at work by high-speed atomic force microscopy

**DOI:** 10.1126/sciadv.adh1069

**Published:** 2023-06-30

**Authors:** Shotaro Tsujioka, Ayumi Sumino, Yutaro Nagasawa, Takashi Sumikama, Holger Flechsig, Leonardo Puppulin, Takuya Tomita, Yudai Baba, Takahiro Kakuta, Tomoki Ogoshi, Kenichi Umeda, Noriyuki Kodera, Hideji Murakoshi, Mikihiro Shibata

**Affiliations:** ^1^ Institute for Frontier Science Initiative, Kanazawa University, Kanazawa, Ishikawa 920-1192, Japan.; ^2^ WPI Nano Life Science Institute (WPI-NanoLSI), Kanazawa University, Kanazawa, Ishikawa 920-1192, Japan.; ^3^ Department of Physiological Sciences, The Graduate University for Advanced Studies, Hayama, Kanagawa 240-0193, Japan.; ^4^ Supportive Center for Brain Research, National Institute for Physiological Sciences, Okazaki, Aichi 444-8585, Japan.; ^5^ Graduate School of Natural Science and Technology, Kanazawa University, Kanazawa Ishikawa 920-1192, Japan.; ^6^ Department of Synthetic Chemistry and Biological Chemistry, Graduate School of Engineering, Kyoto University, Kyoto, Kyoto 615-8510, Japan.

## Abstract

Ca^2+^/calmodulin-dependent protein kinase II (CaMKII) plays a pivotal role in synaptic plasticity. It is a dodecameric serine/threonine kinase that has been highly conserved across metazoans for over a million years. Despite the extensive knowledge of the mechanisms underlying CaMKII activation, its behavior at the molecular level has remained unobserved. In this study, we used high-speed atomic force microscopy to visualize the activity-dependent structural dynamics of rat/hydra/*C. elegans* CaMKII with nanometer resolution. Our imaging results revealed that the dynamic behavior is dependent on CaM binding and subsequent pT286 phosphorylation. Among the species studies, only rat CaMKIIα with pT286/pT305/pT306 exhibited kinase domain oligomerization. Furthermore, we revealed that the sensitivity of CaMKII to PP2A in the three species differs, with rat, *C. elegans*, and hydra being less dephosphorylated in that order. The evolutionarily acquired features of mammalian CaMKIIα-specific structural arrangement and phosphatase tolerance may differentiate neuronal function between mammals and other species.

## Teaser

High-speed atomic force microscopy reveals the activity-dependent structural dynamics of rat/hydra/C. elegans CaMKII

## INTRODUCTION

Ca^2+^/calmodulin-dependent protein kinase II (CaMKII) is a serine/threonine kinase with essential roles in various neuronal cell functions, such as long-term potentiation (LTP), long-term depression (LTD), learning and memory (*1*–*5*). Among four mammalian CaMKII variants (α, β, γ, and δ), α is the most abundantly expressed in the forebrain (*6*, *7*). Notably, in the hippocampus, CaMKIIα represents ~2% of total proteins by mass (*8*) and is further concentrated (~7%) in the postsynaptic density (PSD) fraction (*9*).

**Fig. 1.**
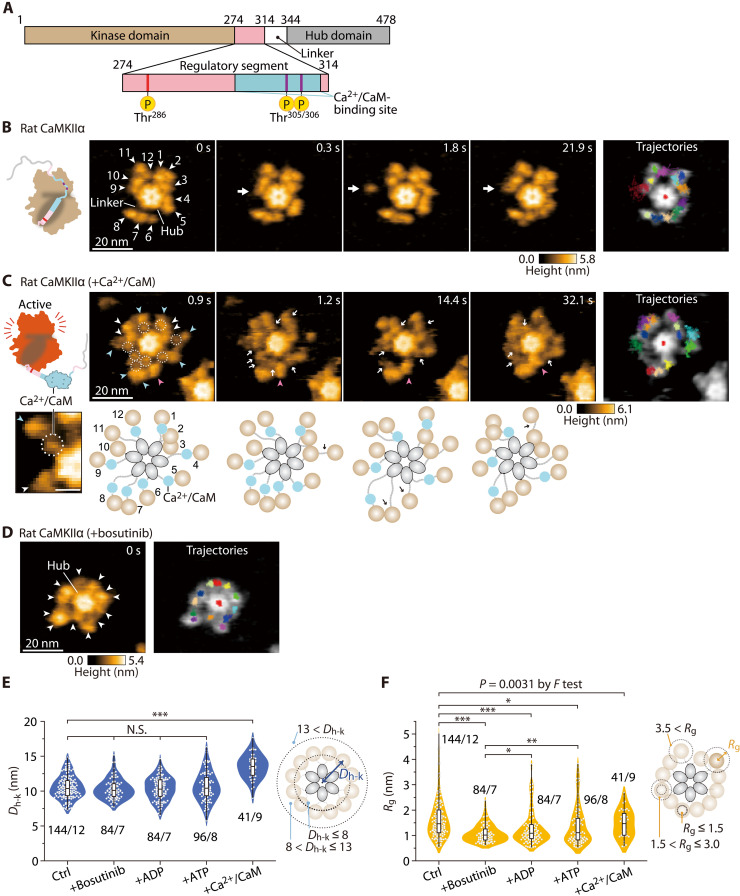
Ca^2+^/CaM binding causes extension of the CaMKIIα holoenzyme. (**A**) Domain structure of rat CaMKIIα. Numbers correspond to the amino acid sequence. (**B**) Sequential HS-AFM images of rat CaMKIIα (Ctrl; see also movie S1A). White arrowheads indicate kinase domains with arbitrary numbers. White arrows indicate the kinase domains in motion. Frame rate, 3.3 frames/s [also in (C) and (D)]. Trajectories of the kinase domains and the center of the hub assembly (red in the center) were tracked for ~30 s [also in (C) and (D)]. (**C**) Sequential HS-AFM images of Ca^2+^/CaM-bound rat CaMKIIα (1 mM Ca^2+^ and 800 nM CaM; see also movie S2B). The dotted circles and white arrows indicate Ca^2+^/CaM. Blue and magenta arrowheads indicate Ca^2+^/CaM bound and dissociating kinase domains, respectively. Diagrams of the presumed structures of the linker regions in 12meric CaMKIIα are also shown at the bottom of each HS-AFM image. (**D**) HS-AFM images of rat CaMKIIα in the presence of 50 μM bosutinib (see also movie S2A). White arrowheads indicate kinase domains. (**E** and **F**) *D*_h-k_ (E) and *R*_g_ (F) for the respective conditions. The number of samples (kinases/holoenzymes) is indicated in the figure. Right: Illustrations of *D*_h-k_ and *R*_g_. “+Ca^2+^/CaM” means surely CaM-bound kinase domains. *P < 0.05, **P < 0.01, and ***P < 0.001 (Kruskal-Wallis test with Dunnett’s post hoc test). (F) Independent Dunnett’s test was performed with Ctrl and +Bosutinib as controls. To compare variances, *F* test was performed between Ctrl and +Ca^2+^/CaM. HS-AFM experiments were repeated at least three times independently with similar results.

CaMKIIα consists of four components: a kinase domain, a regulatory segment with a Ca^2+^/CaM-binding site and three major phosphorylation sites (Thr^286^, Thr^305^, and Thr^306^), a linker region (residues 315 to 344), and a hub domain (Fig. 1A) (*4*). The structural features of the kinase domain, the regulatory segment, and the hub domain are well conserved across metazoans such as the rat, hydra, and *Caenorhabditis elegans* (fig. S1A). In contrast, the linker region is relatively variable among these three species, implying that the linker is possibly involved in distinct activation mechanisms and functions among species.

Mammalian CaMKIIα forms a double-ring structure consisting of 12 subunits connected via a hub domain (fig. S2A) (*1*, *10*). Recently, single-particle electron microscopy showed that the CaMKIIα holoenzyme in the basal state adopts an extended conformation (*11*). In addition, while the kinase domain positioning is highly diverse, each kinase domain is autoinhibited by its regulatory segment (fig. S2B). This autoinhibition is released by the binding of activated CaM (Ca^2+^/CaM) to the regulatory segment, leading to kinase activation (fig. S2B). The adjacent activated kinase domains autophosphorylate each other at Thr^286^ (pT286). Even after Ca^2+^/CaM dissociation, the CaMKIIα subunit maintains pT286 and functions in adjacent kinase phosphorylation. This CaMKIIα autonomy has been hypothesized to be a form of molecular memory (as autonomy enables CaMKIIα to “remember” past Ca^2+^ stimuli by sustained activity) (*1*–*5*).

Upon pT286 and subsequent Ca^2+^/CaM dissociation, the secondary phosphorylation sites (Thr^305/306^) are exposed and autophosphorylated by both inter- (trans) and intramolecular (cis) subunits (fig. S2B) (*12*–*14*). Phosphorylation at Thr^305/306^ (pT305/pT306) inhibits subsequent Ca^2+^/CaM binding (*15*) and decreases the affinity for PSD (*16*, *17*). These characteristics are associated with synaptic plasticities, such as LTP and LTD (*14*, *18*). Specifically, some reports show that pT286 is required for LTP (*19*) and LTD (*20*). Furthermore, a recent study has implicated that the presence or absence of additional pT305/pT306 determines the direction of plasticity (*14*). These results suggest that the combination of Thr^286^ and Thr^305/306^ autophosphorylations within the CaMKII holoenzyme may ultimately determine the outcome of LTP and LTD.

While accumulated evidence has revealed the activation mechanisms and functions of CaMKII in synaptic plasticity, no one has previously observed the structural dynamics of CaMKII in liquid at the molecular level. Here, using high-speed atomic force microscopy (HS-AFM) (*21*–*23*), we visualized the activity-dependent structural dynamics of CaMKII in various states and species, including rat, hydra, and *C. elegans*, in real-time at nanometer resolution.

## RESULTS

### The kinase domains in the CaMKIIα holoenzyme move freely around the hub assembly

For HS-AFM observation, CaMKIIα holoenzymes in the absence of adenosine diphosphate (ADP)/adenosine triphosphate (ATP) were purified from human embryonic kidney (HEK) 293 cells with two-step purification using His- and Strep-tags (fig. S3). Their competency for autophosphorylation at Thr^286^ and Thr^305/306^ was confirmed by Western blotting (fig. S4). The purified proteins were immobilized on a positively charged mica surface modified with pillar[5]arene (*24*). This procedure enabled the visualization of single-particle dynamics of the CaMKIIα holoenzyme at high spatiotemporal resolution (<1 nm, 0.3 s/frame). In HS-AFM videos, individual particles appeared to consist of 12-mers with a central hub assembly and peripheral kinase domains in motion [more than 97% were 12-mer and 3% were 14-mer (*n* = 96); Fig. 1, B to D, and movie S1A], consistent with the previous study (*11*). To analyze the motion of kinase domains, we focused on CaMKIIα holoenzyme with 12-mers, and the position of individual kinase domains in HS-AFM images was traced (Materials and Methods). Principal components analysis (PCA), which detected the interlocking motion of the 12 kinase domains, revealed that the main movement was circumferential around the central hub assembly (fig. S5). To quantify the mobility of each individual kinase domain, we determined the distance between the kinase domain and the center of the hub assembly (*D*_h-k_) and the radius of gyration *R*_g_. In particular, *R*_g_ was calculated as the root mean square displacement of the kinase domain position from its average position, indicating the compactness of the movement range of the individual kinase domains (Materials and Methods). The results showed that each kinase domain was located at an average distance of 10.57 ± 0.36 nm from the center of the hub assembly with a large distribution of *R*_g_ [1.67 ± 0.25 nm (±SD); Ctrl in Fig. 1 (E and F)], consistent with a previous study by electron microscopy (*11*). HS-AFM movie and analysis clearly showed that individual kinase domains are highly mobile.

Previous studies have shown that the linker length between the regulatory segment and the hub domain determines the size of the CaMKII holoenzyme (*25*). Consistent with this, our HS-AFM observations of the no-linker rat CaMKIIα (nlCaMKIIα; lacking amino acids 315 to 344; fig. S1A) showed a more compact form than the wild type (fig. S6 and movie S1). The distribution of *R*_g_ of nlCaMKIIα was narrower than that of the wild type, possibly attributed to the interplay between the kinase and hub domains. Conversely, nlCaMKIIα_I321E_, harboring a point mutation (Ile^321^ to Glu) in the hub domain, corresponding to I351E in the full-length rat sequence, which attenuates the kinase-hub interaction, exhibited analogous *D*_h-k_ and *R*_g_ distribution to the wild type, as depicted in fig. S6. This finding supports the small-angle X-ray scattering (SAXS) experiment (*25*). Furthermore, the resemblance of *R*_g_ values of nlCaMKIIα_I321E_ to the full-length wild type possibly signifies the absence or marginal presence of kinase-hub interactions in the latter under our experimental conditions. Both nlCaMKIIα and nlCaMKIIα_I321E_ did not exhibit Thr^286^ autophosphorylation in our experimental condition, as shown in fig. S4.

Next, we observed the CaMKII holoenzyme in the presence of bosutinib (SKI-606), an ATP-competitive inhibitor (*26*). Bosutinib was initially developed as a protein tyrosine kinase inhibitor (*27*) but was later discovered to also bind to CaMKII in an ATP-competitive manner (*25*, *28*). In the presence of bosutinib, *D*_h-k_ was comparable to that of CaMKIIα (Ctrl), consistent with a prior study demonstrating that bosutinib binding does not alter the size of the nlCaMKIIα holoenzyme (*25*). However, kinase motion (*R*_g_) was more restricted than in Ctrl (Fig. 1, D to F, and movie S2B). This finding indicates the potential modulation of the kinase domain’s conformational dynamics triggered by the interaction of bosutinib with the ATP-binding pocket. This structural alteration in the kinase domain is likely to promote a higher binding affinity toward the regulatory segment. *R*_g_ in bosutinib-bound CaMKIIα is smaller than those in nucleotide-bound CaMKIIα, suggesting that bosutinib binding forms a more robust interaction.

To further investigate, we monitored Ca^2+^/CaM binding to CaMKIIα by measuring Förster resonance energy transfer (FRET) by two-photon fluorescence lifetime imaging microscopy (2pFLIM) (*29*, *30*). Monomeric EGFP (mEGFP)–fused CaMKIIα and mCherry-fused CaM were cotransfected into HeLa cells, and calcium ion influx into the cells was induced by ionophore bath application to activate CaM in the absence or presence of bosutinib (fig. S7). The results showed that bosutinib significantly reduced the efficiency of FRET, suggesting that Ca^2+^/CaM binding to CaMKIIα was predominantly inhibited (fig. S7, B and C). Because bosutinib binding significantly suppresses the kinase domain mobility compared to ADP/ATP-bound states (Fig. 1F), bosutinib-bound CaMKIIα may exhibit tight binding between the kinase domain and the regulatory segment and inhibit Ca^2+^/CaM binding. Therefore, we speculate that the inhibition of Ca^2+^/CaM binding is due to the limited accessibility caused by increased interaction between the kinase domain and the regulatory segment.

### Ca^2+^/CaM binding causes further extension of the CaMKIIα holoenzyme

To visualize the Ca^2+^/CaM-bound CaMKIIα holoenzymes, we preincubated them for 5 to 20 min before performing HS-AFM observations. We avoided direct Ca^2+^/CaM incubation during HS-AFM observation because it significantly increases the background noise due to nonspecific CaM binding to the mica surface.

First, Ca^2+^/CaM-bound CaMKIIα was visualized in the absence of ATP (i.e., with no phosphorylation). It exhibited a further extended form, estimated to be 2.94 nm more extended than that of Ctrl (Fig. 1, C and E, and movie S2C), probably because of the dissociation of the regulatory segment from the kinase domain. Small spherical objects, most likely Ca^2+^/CaM, were observed between the hub and kinase domains (dotted circles in Fig. 1C and movie S2C). HS-AFM also captured Ca^2+^/CaM dissociation and the concomitant approach of the kinase domain to the hub assembly (magenta arrowheads in Fig. 1C and fig. S8).

The average *R*_g_ (1.47 ± 0.54 nm) of Ca^2+^/CaM-bound CaMKIIα was comparable to that of Ctrl (Fig. 1F), but the highly mobile fraction (*R*_g_ greater than 3 nm) seen in Ctrl significantly disappeared [comparison of Ctrl and +Ca^2+^/CaM by *F* test (*P* = 0.0031), in Fig. 1F], suggesting that the mobility of kinase domains with Ca^2+^/CaM binding was restricted. The restricted kinase domains may facilitate efficient trans-phosphorylation in the oligomer.

### Ca^2+^/CaM-bound CaMKIIα holoenzymes with pT286 exhibit the extended form and have highly mobile kinase domains

**Fig. 2.**
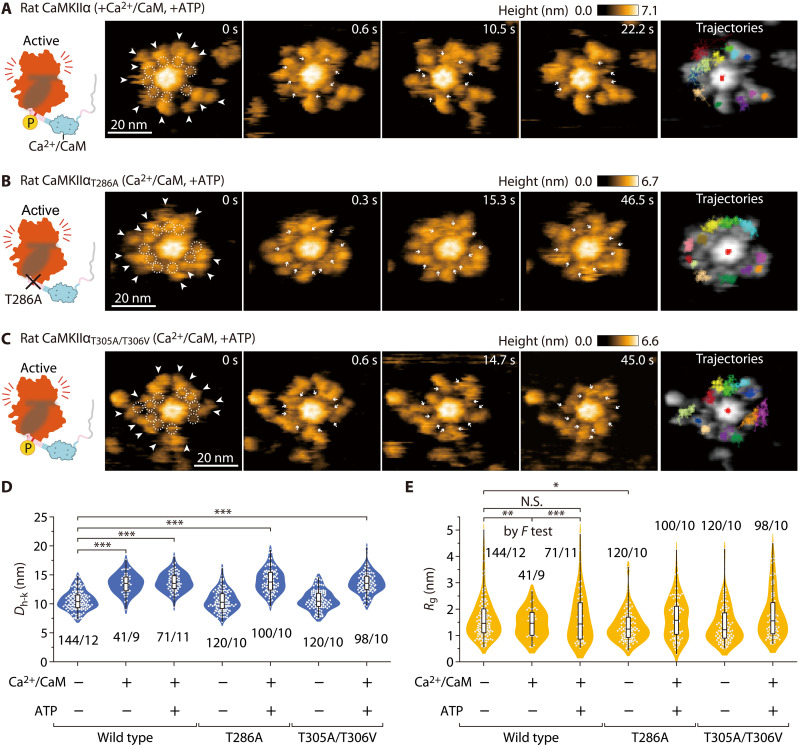
The CaMKIIα holoenzyme with pT286 in the Ca^2+^/CaM-bound state retains its extended structure and high mobility. (**A**) Sequential HS-AFM images (3.3 frames/s) of Ca^2+^/CaM-bound rat CaMKIIα with ATP (see also movie S3). CaMKIIα was activated by Ca^2+^/CaM and ATP (1 mM Ca^2+^, 800 nM CaM, and 1 mM ATP) [also in (B) and (C)]. White arrowheads indicate kinase domains. Dotted white circles and white arrows indicate Ca^2+^/CaM [also in (B) and (C)]. (**B**) Sequential HS-AFM images of Ca^2+^/CaM-bound rat CaMKIIα_T286A_ with ATP (see also movie S4). (**C**) Sequential HS-AFM images of Ca^2+^/CaM-bound rat CaMKIIα_T305A/T306V_ with ATP (see also movie S5). (**D** and **E**) Distances from the center of the hub assembly to kinase domains (*D*_h-k_) (D) and gyration radii *R*_g_ (E) for the respective condition. *P < 0.05 and ***P < 0.001 (Kruskal-Wallis test with Dunnett’s post hoc test). The number of samples (kinases/holoenzymes) is indicated in the figure. (E) To compare variances, *F* test was performed between Ctrl, +Ca^2+^/CaM, and +Ca^2+^/CaM + ATP in the wild type. **P < 0.01; ***P < 0.001; and N.S., not significant. HS-AFM experiments were repeated at least three times independently with similar results.

Next, we preincubated CaMKIIα and Ca^2+^/CaM with ATP to prepare phosphorylated (pT286) CaMKIIα (fig. S4) and observed it under HS-AFM (Fig. 2, A to C, and movie S3). In CaMKIIα (Ca^2+^/CaM and pT286), Ca^2+^/CaM appeared between the hub and the kinase domains (dotted circles in Fig. 2A and movie S3B). Compared to that of nonphosphorylated CaMKIIα, the binding of Ca^2+^/CaM was more stable [the fractions of Ca^2+^/CaM bound longer than 60 s were 77.3% (*n* = 66) and 41.1% (*n* = 56) for pT286 and the nonphosphorylated state, respectively]. This result is consistent with the CaM-trapping model (*31*). It has been reported that under these buffer conditions (1 mM CaCl_2_ and 1 mM ATP), Ca^2+^/CaM can continue to bind to CaMKIIα virtually indefinitely (*31*). However, we often found that Ca^2+^/CaM was released from CaMKIIα during observation. It may be due to the invasive interaction between an AFM tip and Ca^2+^/CaM. Similar to CaMKIIα (Ca^2+^/CaM) (Fig. 2D), CaMKIIα (Ca^2+^/CaM and pT286) showed a structure extended in the radial direction by 3.3 nm. One of the remarkable differences is the reappearance of high *R*_g_ [comparison of +Ca^2+^/CaM and +Ca^2+^/CaM + ATP by *F* test (*P* = 0.00037) in Fig. 2E]. We also imaged Ca^2+^/CaM-bound and Ca^2+^/CaM-unbound states of two different mutants (i.e., CaMKIIα_T286A_ and CaMKIIα_T305A/T306V_; Fig. 2, B to E; figs. S4 and S9; and movies S4 and S5). We found that the structural change after Ca^2+^/CaM binding in the mutants was similar to that observed in the wild type.

### Fully phosphorylated rat CaMKIIα holoenzymes (pT286/pT305/pT306) exhibit internal kinase domain oligomerization

**Fig. 3.**
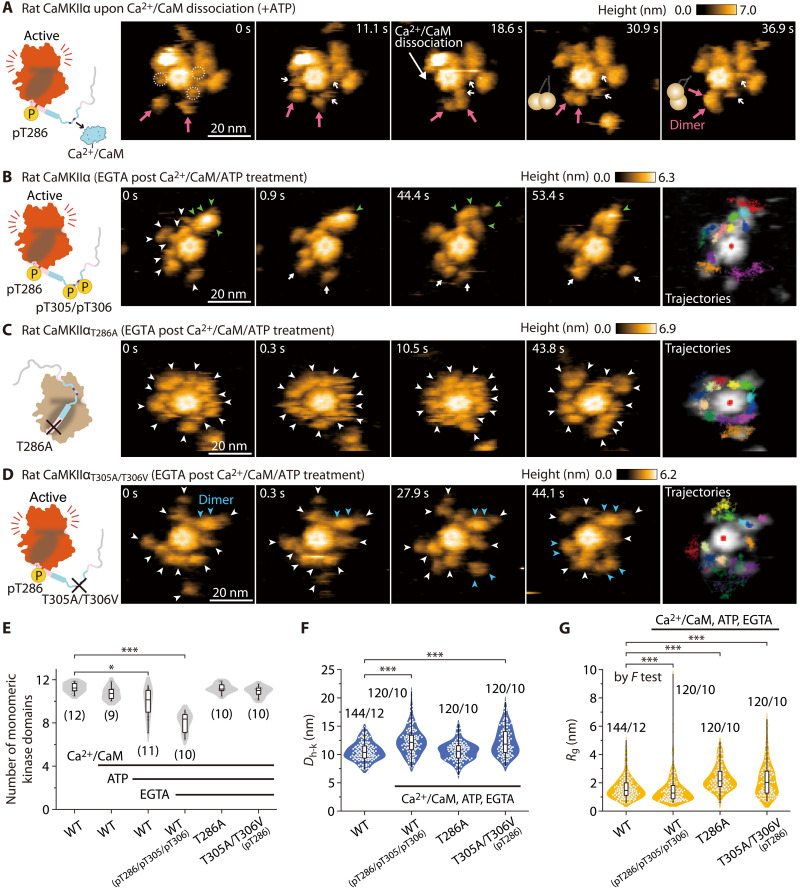
Fully phosphorylated rat CaMKIIα (pT286/pT305/pT306) exhibits KD-oligomerization. (**A**) Sequential HS-AFM images of rat CaMKIIα at the time of stable dimer formation (see also movie S3B). CaMKIIα was activated by Ca^2+^/CaM and ATP (1 mM Ca^2+^, 800 nM CaM, and 1 mM ATP). Dotted white circles and white arrows indicate Ca^2+^/CaM. Magenta arrows indicate the formation of stable dimers. Frame rate, 3.3 frames/s [also in (B) to (D)]. (**B**) Sequential HS-AFM images of pT286/pT305/pT306 rat CaMKIIα. CaMKIIα was first activated to induce pT286, as described in (A). Subsequently, EGTA (2 mM) was added to induce Ca^2+^/CaM dissociation and pT305/pT306 (autophosphorylation) [also in (C) and (D)]. Arrowheads indicate kinase domains (green, oligomer; blue, dimer; white, monomer) [also in (C) and (D)]. Criteria of oligomer are described in Materials and Methods. (**C**) Sequential HS-AFM images of rat CaMKIIα_T286A_ (see also movie S4). This mutant does not autophosphorylate at Thr^286^/Thr^305^/Thr^306^ (fig. S4, lanes #3 to 5). (**D**) Sequential HS-AFM images of pT286 rat CaMKIIα_T305A/T306V_ (see also movie S5). (**E**) The number of monomeric kinase domains (i.e., the 4-nm objects surrounding the hub assembly of CaMKIIα). The number of samples (holoenzymes) is indicated in the figure. *P < 0.05 and ***P < 0.001 (Kruskal-Wallis test with Dunnett’s post hoc test) [also in (F) and (G)]. (**F** and **G**) *D*_h-k_ (F) and *R*_g_ (G) for the respective conditions. The number of samples (kinases/holoenzymes) is indicated in the figure. (G) To compare variances, *F* test was performed between Ctrl and pT286/pT305/pT306 in the wild type. HS-AFM experiments were repeated at least three times independently with similar results.

During HS-AFM observation of CaMKIIα (Ca^2+^/CaM and pT286), we found that the kinase domains formed stable dimers upon Ca^2+^/CaM dissociation (magenta arrows in Fig. 3A and third molecule in movie S3B). To clarify the molecular mechanisms of the stable oligomerization of kinase domains, we prepared Ca^2+^/CaM-unbound and fully phosphorylated CaMKIIα (pT286/pT305/pT306 in fig. S2B). In preparation, we incubated CaMKIIα in the presence of Ca^2+^/CaM and ATP, inducing pT286. Subsequently, Ca^2+^/CaM was released by incubation with an excess of EGTA, causing *trans*/*cis*-phosphorylation at pT305/pT306 (Fig. 3 and fig. S4). In the HS-AFM videos, no Ca^2+^/CaM was observed in the CaMKIIα holoenzyme, indicating that most CaM was dissociated in this experimental condition (Fig. 3, B to D, and movie S3C).

HS-AFM videos revealed the existence of two distinct states of the kinase domains, namely, oligomeric and monomeric states (green and white arrowheads, respectively, in Fig. 3B and movie S3C). To quantify the extent of kinase domain oligomerization (KD-oligomerization), we assessed the number of monomeric kinase domains. Because the kinase domain’s diameter is typically 4 to 5 nm, we postulated that protrusions measuring around 4 nm in diameter around the central hub assembly corresponded to the monomeric kinase domain (see Materials and Methods). The analysis showed a significant reduction in the apparent kinase domains in the fully phosphorylated CaMKIIα sample, possibly because of increased kinase domain oligomers (Fig. 3E).

The kinase domains (pT286/pT305/pT306) showed larger *D*_h-k_ (Fig. 3F) and broader distribution of *R*_g_ compared with Ctrl (*P* < 0.0001 by *F* test in Fig. 3G). The broad distribution is due to the presence of two types of kinase domains: monomers with high mobility and oligomers with less mobility. To conduct a further control experiment, ADP was added instead of ATP, and it was confirmed that neither structural change nor KD-oligomerization occurred (fig. S10).

We next investigated the dependence of pT286 and pT305/pT306 on KD-oligomerization by using CaMKIIα_T286A_ and CaMKIIα_T305A/T306V_ mutants. The results indicated that CaMKIIα_T286A_ did not exhibit KD-oligomerization (Fig. 3, C and E, and movie S4C). Notably, this mutant showed no phosphorylation at Thr286/Thr^305^/Thr^306^ (fig. S4). Furthermore, kinase domains of CaMKIIα_T305A/T306V_ did not also exhibit KD-oligomerization (Fig. 3, D and E, and movie S5C). In contrast to CaMKIIα_T286A_, CaMKIIα_T305A/T306V_ had a longer *D*_h-k_ (12.32 ± 2.76 nm; ±SD in Fig. 3F) and a larger *R*_g_ (2.50 ± 0.36 nm; ±SD in Fig. 3G), supporting the idea that pT286 causes a further extension and mobility on top of the state, which is initially induced by Ca^2+^/CaM. In addition, we found that adjacent subunits of CaMKIIα_T305A/T306V_ temporarily formed a transient dimer (blue arrowheads in Fig. 3D; the dimer formation was determined by the size of the kinase domains in HS-AFM images), but not stable KD-oligomerization. Thus, phosphorylation at both Thr^286^ and Thr^305/306^ is required for stable KD-oligomerization.

### Hydra CaMKIIα and *C. elegans* CaMKII do not exhibit KD-oligomerization in the fully phosphorylated state

**Fig. 4.**
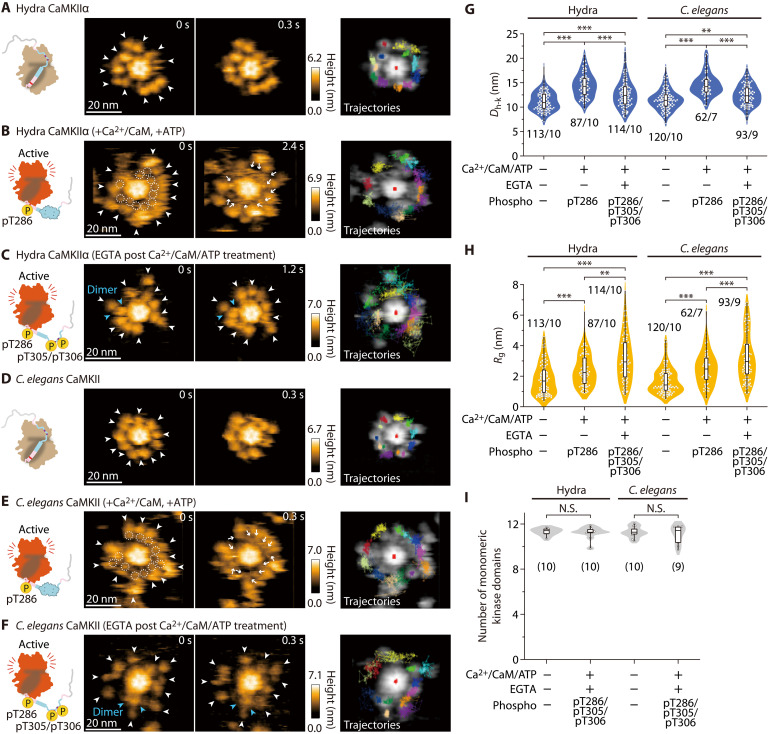
Hydra CaMKIIα and *C. elegans* CaMKII do not exhibit KD-oligomerization. (**A** to **F**) HS-AFM images of hydra and *C. elegans* CaMKII (see also movies S5 and S6). The experimental conditions are described in the figures. Frame rate, 3.3 frames/s. In (B) and (E), Ca^2+^/CaM-bound pT286 CaMKII were prepared by activated CaM and ATP (1 mM Ca^2+^, 800 nM CaM, and 1 mM ATP). In (C) and (F), pT286 CaMKII was further treated with 2 mM EGTA to induce Ca^2+^/CaM dissociation and pT305/pT306 (autophosphorylation). Arrowheads indicate kinase domains (blue, dimer; white, monomer). Criteria of oligomer are described in Materials and Methods. (**G** and **H**) *D*_h-k_ (G) and *R*_g_ (H) for the respective conditions. The number of samples (kinases/holoenzymes) is indicated in the figure. **P < 0.01 and ***P < 0.001 (Kruskal-Wallis test with Dunn’s post hoc test). HS-AFM experiments were repeated at least three times independently with similar results [also in (I)]. (**I**) The number of monomeric kinase domains (i.e., the 4-nm objects surrounding the hub assembly of CaMKIIα). The number of samples (holoenzymes) is indicated in the figure. N.S., not significant (Kruskal-Wallis test).

CaMKII is highly conserved across species such as hydra and *C. elegans* (fig. S1A) (*25*). However, are the features of the structural dynamics seen in rat CaMKIIα also observable in other species? To address this question, we used HS-AFM to investigate hydra CaMKIIα and *C. elegans* CaMKII, which are representative of the early stages of animal evolution. Comparable to rat CaMKIIα, the kinase domains of both species exhibited free motion around the central hub assembly in both the inactive and active (Ca^2+^/CaM, pT286) states (Fig. 4, A to F, and movies S6 and S7) and underwent extension upon Ca^2+^/CaM binding (Fig. 4, B, E, and G).

Next, we observed hydra CaMKIIα and *C. elegans* CaMKII in the fully phosphorylated state (pT286/pT305/pT306; fig. S4) and found that those kinase domains were not oligomerized but highly mobile (Fig. 4, C, F, H, and I). These data suggest that KD-oligomerization in CaMKIIα at pT286/pT305/pT306 is mammalian specific.

To determine the biochemical function of KD-oligomerization, we assessed the kinase activity with a CaMKIIα substrate peptide, syntide-2 (*32*), by Western blotting. Rat CaMKIIα (pT286/pT305/pT306), responsible for KD-oligomerization, more efficiently phosphorylated the substrate peptide (lanes #1/2 in fig. S11). A similar result was obtained with CaMKIIα_T305A/T306V_, which does not form KD-oligomerization, suggesting that the increased kinase activity is not due to KD-oligomerization (lanes #3/4 in fig. S11). However, we found a significant reduction in the kinase activity of hydra CaMKIIα and no alteration in that of *C. elegans* CaMKII in the fully phosphorylated state (fig. S11). As these kinase domains remain monomeric with pT286/pT305/pT306 phosphorylation, it suggests that KD-oligomerization does not govern kinase activity. Rather, an unknown mechanism related to the phosphorylation at pT286/pT305/pT306 is likely contributing to the regulation of kinase activity.

**Fig. 5.**
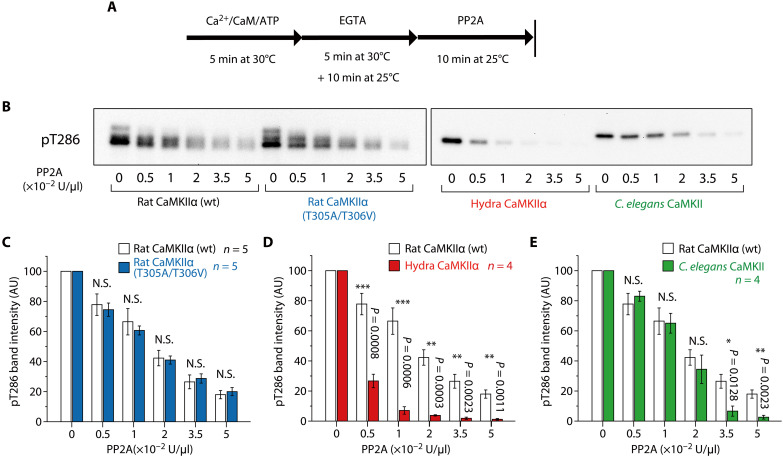
Assay of pT286/pT305/pT306 CaMKII holoenzyme dephosphorylation. (**A** and **B**) Dephosphorylation of CaMKII by PP2A was detected by Western blotting. The time course of the experiment (A) and the representative result of Western blotting (B) are shown. pT286/pT305/pT306 CaMKII was prepared as described in the Materials and Methods. This protocol also induces KD-oligomerization in rat CaMKIIα holoenzymes (Fig. 3B). Subsequently, the indicated amount of PP2A was incubated for 10 min at 25°C. (**C** to **E**) Statistical comparison of pT286 band intensities of rat CaMKIIα (wt) and the T305A/T306V mutant (C), hydra CaMKIIα (D), and *C. elegans* CaMKII (E). In rat CaMKIIα bands (B), there was a slight smearing, so the entire region, including the smeared region, was measured. Error bars indicate SEMs, and the number of experiments is shown in the figures. N.S. (not significant, *P* > 0.05); *P < 0.05; and ***P < 0.001; *t* test. AU, arbitrary units.

Next, we sought to determine whether KD-oligomerization in rat CaMKIIα (pT286/pT305/pT306) confers resistance to dephosphorylation. We incubated phosphorylated CaMKIIα with protein phosphatase 2A (PP2A), a major CaMKII phosphatase (*16*, *33*), and quantified phosphorylation at Thr^286^ by Western blotting (Fig. 5, A and B). In the absence of PP2A, the band intensities were comparable among species (fig. 12). Both phosphorylated wild-type rat CaMKIIα and its non–KD-oligomerizing mutant (T305A/T306V) were found to be similarly dephosphorylated in a concentration-dependent manner by PP2A (Fig. 5C), suggesting that KD-oligomerization does not confer greater resistance to PP2A. In contrast, we found that rat CaMKIIα is more resistant to PP2A than hydra CaMKIIα over a broad concentration range (Fig. 5D). Rat CaMKIIα was also more resistant than *C. elegans* CaMKII at high PP2A concentrations (Fig. 5E).

## DISCUSSION

**Fig. 6.**
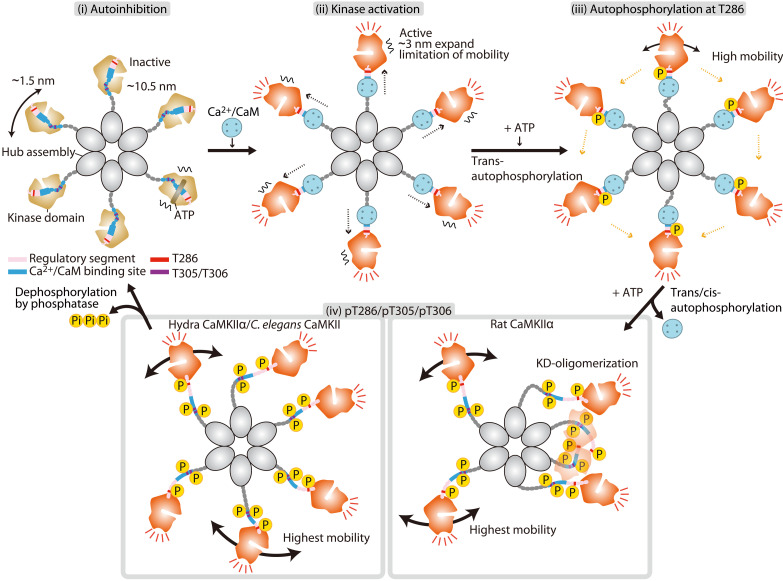
Schematic of CaMKIIα dynamics in the process of activation. A dodecameric CaMKIIα holoenzyme is depicted as a hexamer for simplicity. (i) In an inactive state, kinase domains are distributed within ~10.5 nm in radius from the center of the hub assembly with some mobility. (ii) Ca^2+^/CaM binding leads to the extended form of the holoenzyme and mobility reduction of kinase domains. (iii) Subsequent Thr^286^ autophosphorylation (autophosphorylation at T286) leads to higher kinase mobility. (iv) Dissociation of Ca^2+^/CaM induces an increase in kinase mobility (highest mobility) and causes autophosphorylation at Thr^305^/Thr^306^. The pT286/pT305/pT306 state of rat CaMKIIα exhibits KD-oligomerization, while hydra CaMKIIα and *C. elegans* CaMKII do not. The phosphorylated state is maintained until dephosphorylation by phosphatase occurs.

CaMKII has been extensively investigated by various methods such as biochemical assays and electron microscopy. However, the visualization of single-molecule CaMKII holoenzymes in action has not been achieved. In this study, we used HS-AFM to observe the activity-dependent structural dynamics of CaMKII from different species, including rats, hydras, and *C. elegans*. Building upon the analyses of our observations, we described (i) the effects of ADP/ATP/bosutinib binding, (ii) the restriction of movement of kinase domains upon Ca^2+^/CaM binding, (iii) the autophosphorylation-dependent KD-oligomerization in mammalian CaMKIIα but not in older nonmammalian species, and (iv) the higher phosphatase tolerance of mammalian CaMKIIα compared to nonmammalian species. On the basis of our HS-AFM results, we propose a CaMKII activation mechanism (Fig. 6). In addition, HS-AFM revealed that CaMKII has more complex species-dependent conformational states and functions than previously anticipated.

Previously, 3D reconstruction from electron microscopy with pseudoatomic resolution demonstrated that the CaMKII holoenzyme, in its basal state, adopts an extended conformation and exhibits high flexibility in the positioning of its kinase domain, and the majority exists as dodecamers (*11*). Consistent with this observation, we found that the kinase domains in the CaMKIIα holoenzyme, in the basal state, exist in a mobile state, likely facilitating the binding of Ca^2+^/CaM to CaMKIIα. While the existence of kinase domain dimers was predicted (*11*, *34*), we observed that most domains existed as monomers. This discrepancy could be attributed to methodological differences between the studies.

We assessed the structure of the rat CaMKIIα holoenzyme in the presence and absence of bosutinib. Consistent with a previous study (*25*), we did not detect any alterations in the size of rat CaMKIIα. However, our observations revealed that bosutinib binding restricts the motion of the kinase domain. This restriction could be attributed to an increased affinity between the kinase domain and the regulatory segment, which may hinder Ca^2+^/CaM binding. In addition, 2pFLIM analysis indicated that bosutinib inhibits Ca^2+^/CaM binding to CaMKIIα in HeLa cells. On the basis of these findings, it is likely that bosutinib binding inhibits CaMKIIα activation via two pathways: by competitively obstructing ATP binding and by impeding Ca^2+^/CaM binding by steric hindrance.

Upon Ca^2+^/CaM binding, the CaMKIIα holoenzymes extend by approximately 3 nm from the center of the hub assembly, potentially dissociating the regulatory segment from the active site in the kinase domain and exposing the Thr^286^ phosphorylation site in the regulatory segment, as previously suggested (*35*). The mobility of the kinase domain decreases upon Ca^2+^/CaM binding, but it increases again with pT286, even when Ca^2+^/CaM binding is present. On the basis of the previous report (*35*), we hypothesize that Ca^2+^/CaM binding stabilizes the α-helical structure of the regulatory segment and increases the accessibility of adjacent kinase domains to Thr^286^. Thr^286^ phosphorylation induces a structural transition of the regulatory segment from α-helical to disordered, leading to increased mobility of the kinase domain. This pT286-dependent structural transition may be supported by earlier findings demonstrating that pT286 significantly enhances the affinity to Ca^2+^/CaM (CaM-trap) (*31*).

After Ca^2+^/CaM dissociation, the regulatory segment with pT286 remains free from the active site, and the second phosphorylation site (Thr^305/306^) undergoes trans/cis-phosphorylation (*13*, *14*). Our HS-AFM observations showed that the rat CaMKIIα holoenzyme with pT286/pT305/pT306 phosphorylation displays internal KD-oligomerization. Therefore, we propose that pT286/pT305/pT306 phosphorylation disrupts the structure of regulatory segment, leading to a disordered and entangled string-like configuration, as seen in the microtubule-associated protein tau (*36*). This arrangement could potentially increase the interaction between inter-regulatory segments.

Several potential physiological implications of KD-oligomerization in rat CaMKIIα can be considered. First, KD-oligomerization may affect kinase activity. A kinase assay demonstrated that the CaMKIIα holoenzyme with pT286/pT305/pT306 phosphorylation more efficiently phosphorylates its substrate. This result does not contradict a previous study showing Thr^305/306^-dependent substrate phosphorylation (*14*). However, the non–KD-oligomerizing mutant (i.e., T305A/T306V) also exhibited increased kinase activity, suggesting that KD-oligomerization might not be the direct cause of elevated kinase activity.

Secondly, KD-oligomerization could potentially restrict the accessibility of phosphatases to pT286/pT305/pT306 by sequestering them in the oligomers. However, it is unlikely because oligomeric and monomeric forms of CaMKIIα displayed similar phosphatase tolerance.

Third, KD-oligomerization may regulate the affinity between CaMKIIα holoenzyme and its binding partners. Prior studies have shown that pT305/pT306 decreases affinity with PSD in vitro (*16*), while introducing T305A/T306A enhances association with PSD. This increased association reduces the threshold for hippocampal LTP, resulting in more inflexible and less finely-tuned hippocampal-dependent learning (*18*). Moreover, a recent study has proposed that LTD stimulation phosphorylates Thr^305^/Thr^306^ over Thr^286^ and encourages CaMKII translocation into inhibitory synapses (*14*). In our study, we observed distinct structural variations within the CaMKIIα oligomer between the pT286 and pT286/pT305/pT306 states. These structural variations stemming from different phosphorylations may be responsible for inducing opposing cellular functions such as LTP and LTD.

CaMKII is a protein that has been conserved throughout evolution (*37*). However, the linker region of this protein is hypervariable among different species. To date, the effect of these differences on the dynamics and functions of CaMKII has not been explored. In this study, we used HS-AFM to visualize the structural and dynamic distinctions of rat, hydra, and *C. elegans* CaMKII holoenzymes in liquid. Our results showed that the kinase domains of all three species exist as monomers in the basal and Ca^2+^/CaM-bound pT286 state. In contrast, previously, crystal structural analyses combined with SAXS measurements suggested that the *C. elegans* kinase domain forms dimers (*34*). This discrepancy could be attributed to differences in methodology. The main variation among CaMKIIα species was observed in their pT286/pT305/pT306. In rat CaMKIIα, kinase domains were found to oligomerize, whereas ancestral CaMKII (i.e., hydra and *C. elegans* CaMKII) did not. The dissimilarity in amino acid sequences of the regulatory segment and linker region among species may account for the variance in KD-oligomerization.

One of the notable discoveries of this investigation is the species-specific variation in phosphatase tolerance of pT286/pT305/pT306. Biochemical assays revealed that rat CaMKIIα has greater resistance to PP2A than hydra CaMKIIα and *C. elegans* CaMKII. The cause for the higher phosphatase tolerance remains unknown. However, the discrepancy in *R*_g_ is a plausible explanation for this variation. As *the R*_g_ of nonmammalian CaMKII is larger than that of rat CaMKIIα (fig. S13), nonmammalian CaMKII could have more free space around phosphorylation sites. It could facilitate phosphatase accessibility to phosphorylated regulatory segments for dephosphorylation.

In conclusion, our findings provide a basis for a deeper understanding of the molecular mechanisms of CaMKIIα activation. Specifically, mammalian CaMKIIα-specific structural arrangement and phosphatase tolerance may be critical features for integrating Ca^2+^ signals and sustaining CaMKIIα activation required for LTP, LTD, learning, and memory. Furthermore, these evolutionarily acquired features may distinguish neuronal function between mammals and other species.

## MATERIALS AND METHODS

### Reagent

A calcium ionophore (4-bromo A-23187) was purchased from Cayman Chemical (USA). Bosutinib (catalog no. 4361) was purchased from Tocris (United Kingdom).

### Plasmids

The rat (*Rattus norvegicus*) CaMKIIα gene encoding CaMKIIα (amino acids 1 to 478) was a gift from Y. Hayashi at Kyoto University. The synthesized gene encoding rat (*Rattus norvegicus*) CaM was purchased from Eurofins (Tokyo, Japan). The synthesized genes encoding hydra (*Hydra vulgaris*) CaMKIIα isoform X2 and *C. elegans* CaMKII were purchased from GenScript (Tokyo, Japan). For PP2A-related genes (*38*), human PPP2CA was purchased from Promega (WI, USA), and synthesized human PTPA (PPP2R4) and Mini-A (PPP2R1A) were purchased from FASMAC (Kanagawa, Japan).

The plasmid containing 6×His/Strep tags (MDYKDDDDHHHHHHKWSHPQFEKGTGGQQMGRDLYDDDDKDLYKSGLRSRA) fused to the N terminus of rat/hydra/*C. elegans* CaMKII/PPP2CA/PTPA/Mini-A was inserted into a modified pEGFP-C1 mammalian expression vector (the kanamycin resistance gene was replaced with the ampicillin resistance gene) by replacing EGFP. To construct the no-linker CaMKIIα (nlCaMKIIα) plasmid, the sequence encoding amino acids 315 to 344 was removed from the rat CaMKIIα sequence. CaMKIIα mutants with point mutations were constructed using the QuikChange site-directed mutagenesis kit (Agilent Technologies, USA). To construct 6×His-tagged CaM and superfolder GFP (sfGFP)–fused syntide-2 peptide (amino acid sequence PLARTLSVAGLPGKK) plasmids, the respective genes were inserted into the pRSET bacterial expression vector (Invitrogen, USA). mEGFP-CaMKIIα and mCherry-CaM were constructed by insertion into the modified pEGFP-C1 vector, replacing EGFP.

### Protein purification from bacteria

The His-tagged CaM and sfGFP-fused Syntide-2 were overexpressed in *Escherichia coli* (DH5α), and the culture medium (~250 ml) was centrifuged at 2500*g* for 20 min. The cell pellet was dissolved in 10 ml of ice-cold phosphate-buffered saline (PBS) buffer with 1% Triton X-100 and 5 mM imidazole, sonicated, and then centrifuged at 20,000*g* for 10 min. The supernatant was loaded into a Ni^+^-nitrilotriacetate (NTA) column (HiTrap, GE Healthcare, USA). The NTA column bound to the protein was washed with 5 and 50 mM imidazole containing 20 mM Tris-HCl (pH 7.4) buffer and eluted with 20 mM tris-HCl/500 mM imidazole buffer. The concentration of the purified protein was measured using the Bradford assay (Bio-Rad, USA) by comparing BSA as a standard.

### Protein purification from HEK293 cells

All CaMKII proteins and PP2A were prepared with the following method. 6×His/Strep tagged CaMKIIα was expressed in HEK293 cells. Briefly, HEK293 cell culture was maintained on 15-cm plates in Dulbecco’s modified Eagle’s medium (DMEM) supplemented with 5% fetal bovine serum with no antibiotics at 37°C and 5% CO_2_. Three 15-cm dishes at 70% confluency were prepared for transfection. The cells were transfected with the plasmid-encoding CaMKII genes using Lipofectamine 2000 following the manufacturer’s protocol (Thermo Fisher Scientific, USA). For PP2A, three plasmids encoding PPP2CA/PTPA/Mini-A genes were cotransfected. One day after transfection, the cells were collected and lysed with 10 ml of ice-cold PBS buffer with 1% Triton X-100 and 5 mM imidazole and then centrifuged at 20,000*g* for 10 min. The supernatant was loaded into Strep-Tactin Sepharose (Nacalai, Japan), washed with 20 mM tris-HCl/150 mM KCl, and subsequently eluted with 2.5 mM desthiobiotin (pH 7.4). The pooled proteins were further purified with an NTA column, and the concentration of CaMKII proteins was determined as described above. The PP2A concentration was determined as described in the following section. The purified proteins were stored in 20 mM tris-HCl/150 mM KCl with 50% glycerol and 2 mM dithiothreitol at −30°C before experiments.

### Measurement of purified PP2A activity

One unit is defined as the amount of PP2A that hydrolyzes 1 nmol of 50 mM *p*-nitrophenyl phosphate (pNPP) in 1 min at RT in a total reaction volume of 100 μl. The phosphatase activity was assayed in 100 μl of the reaction mixture [50 mM pNPP, 50 mM tris (pH 7.6), 150 mM KCl, 10 mM MgCl_2_, and 1 mM MnCl_2_]. The reaction was initiated by the addition of 1 μl of PP2A. After 3 min, the amount of *p*-nitrophenol was determined by reading the absorbance at 405 nm (18,000 M^−1^ cm^−1^). This method gives PP2A activity in units per microliter, and our purified PP2A typically had the activity of ~0.5 unit/μl.

### Biochemical assay for phosphorylation/dephosphorylation of CaMKII

Standard kinase assays were performed at 30°C for 5-min incubation with 50 nM purified CaMKII proteins, 800 nM CaM, 1 mM CaCl_2_, and 1 mM ATP in a reaction buffer [50 mM tris-HCl (pH 7.4), 150 mM KCl, and 10 mM MgCl_2_]. To release CaM from CaMKII, 2 mM EGTA was added and incubated for 5 min at 30°C and an additional 10 min at 25°C to match the conditions for AFM observation (i.e., after the sample was loaded on the AFM apparatus, it typically takes 10 min before observation). For substrate phosphorylation, sfGFP-Syn2 was added at 1 μM and incubated for 7 min at 25°C. For CaMKII dephosphorylation by PP2A, phosphorylated CaMKII proteins were incubated with the indicated concentration of PP2A for 10 min at 25°C. The reactions were stopped by adding SDS sample buffer and then analyzed by Western blotting.

Western blotting was performed with the following antibodies: anti–phospho-CaMKII (Thr^286^) (D21E4; Cell Signaling Technology, USA), anti-pT305 (abx012403; Abbexa), anti–phospho-CaMKII (Thr^306^) (p1005-306; PhosphoSolutions, USA), anti–phospho-PKA substrate (RRXS*/T*) (#9624; Cell Signaling Technology), anti–His-tag (27E8; Cell Signaling Technology, USA), and HRP–anti-rabbit/mouse (Jackson Laboratory, USA).

### 2pFLIM-FRET experiment

HeLa cells were cultured in DMEM supplemented with 5% fetal bovine serum at 37°C and 5% CO_2_. The cells were transfected with the plasmids (i.e., mEGFP-CaMKIIα and mCherry-CaM) by means of Avalanche-Everyday transfection reagent (EZ Biosystems, USA), followed by incubation for 16 to 20 hours. FLIM-FRET imaging was conducted in a solution containing Hepes (30 mM, pH 7.3)–buffered artificial cerebrospinal fluid (130 mM NaCl, 2.5 mM KCl, 1 mM CaCl_2_, 1 mM MgCl_2_, 1.25 mM NaH_2_PO_4_, and 25 mM glucose) at room temperature (23° to 25°C).

Details of the 2pFLIM-FRET imaging are described elsewhere (*30*, *39*). Briefly, mEGFP-CaMKIIα was excited with a Ti: sapphire laser (Mai Tai; Spectra-Physics, USA) tuned to 920 nm. The scanning mirror was controlled with ScanImage software (*40*). The green fluorescence photon signals were collected by an objective lens (60×, 1.0 numerical aperture; Olympus, Japan) and detected by a photomultiplier tube (H7422-40p; Hamamatsu, Japan) placed after a dichroic mirror (565DCLP; Chroma Technology, USA) and emission filter (FF01-510/84; Semrock, USA). Fluorescence lifetime measurement was processed using a time-correlated single-photon counting board (SPC-150; Becker & Hickl, Germany) controlled with custom software (*39*). For fluorescence lifetime image construction, the mean fluorescence lifetime in each pixel was translated into a color-coded image (*30*). Analysis of the lifetime change and binding-fraction change was conducted as described elsewhere (*30*).

### HS-AFM apparatus

HS-AFM observations were performed using a homemade high-speed atomic force microscope (*23*, *41*, *42*). An optical beam deflection detector was used to detect the cantilever (BL-AC10DS-A2, Olympus, Japan) deflection in tapping mode using an infrared (IR) laser at 780 nm and 0.7 mW. The IR beam was focused onto the back of the cantilever through a 50× objective lens (TU Plan Apo EPI 50X, Nikon, Japan). The reflection of the IR beam from the cantilever was detected with a two-segmented PIN photodiode (MPR-1, Graviton, Japan). The spring constant of the cantilever was ~100 pN/nm. The resonant frequency and quality factor of the cantilever in a liquid were ~400 kHz and ~2, respectively. Although the cantilever has the original bird beak–like triangular portion as an AFM tip, to improve the spatial resolution of HS-AFM, an amorphous carbon tip was grown on the original AFM tip by electron beam deposition using SEM. The length of the additional AFM tip was ~500 nm, and the apex of the tip was ~1 nm in radius after further plasma etching by a plasma cleaner (Tergeo, P.I.C. Scientific, USA). All HS-AFM images were obtained from cantilevers with additional AFM tips. The free oscillation amplitude of the cantilever was under 1 nm, and the set-point amplitude was set to 80% of the free amplitude during HS-AFM observations. To reduce the force between the sample and the AFM tip, a recently developed “only trace imaging” mode was used for HS-AFM scanning (*43*). HS-AFM data were collected using laboratory-developed software based on Visual Basic.NET (Microsoft).

### Substrate for HS-AFM observations

For all HS-AFM observations, we modified a mica surface using cationic C2 pillar[5]arene (P[5]A+) to change the surface charge from negative to positive (*44*). The electrostatic potential map of CaMKIIα shows that the surface charge of the hub assembly is partially negative (*11*), while the surface charge of the kinase domains is partially positive. The mobility of the kinase domains of CaMKIIα was strongly inhibited on negatively charged bare mica due to electrostatic interactions. To prevent the inhibition of kinase domain mobility on an HS-AFM substrate, we used P[5]A+ as an AFM substrate. An aqueous solution of 70 μM P[5]A+ was deposited onto a freshly cleaved mica substrate (1.0 mm in diameter; Furuuchi Chemical, Japan). P[5]A+ was incubated at room temperature on the mica surface for 15 min, and the surface was rinsed with Milli-Q water to remove unadsorbed P[5]A+.

### HS-AFM observations

CaMKII holoenzymes in the basal state were observed in buffer A [50 mM tris-HCl (pH 7.4), 15 mM KCl, 10 mM MgCl_2_, and 10% glycerol] with 0.1 mM EGTA. For the inhibitor experiment, 50 nM CaMKIIα was premixed at 30°C for 5 min with 50 μM bosutinib in buffer B [50 mM tris-HCl (pH 7.4), 150 mM KCl, and 10 mM MgCl_2_] with 0.1 mM EGTA. Then, HS-AFM observations were performed in buffer A with 0.1 mM EGTA and 50 μM bosutinib.

For Ca^2+^/CaM-bound CaMKII holoenzymes, 50 nM CaMKII and 800 nM CaM were premixed in buffer B with 1 mM CaCl_2_ and incubated at 30°C for 5 min. Then, HS-AFM observations were performed in buffer A with 1 mM CaCl_2_.

For Ca^2+^/CaM-bound CaMKII holoenzymes in the presence of ATP, we premixed 50 nM CaMKII and 800 nM CaM in buffer B with 1 mM CaCl_2_ and 1 mM ATP and incubated the mixture at 30°C for 5 min. Then, HS-AFM observations were performed in buffer A with 1 mM CaCl_2_ and 1 mM ATP.

For CaMKII holoenzymes in EGTA and ATP, we premixed 50 nM CaMKII and 800 nM CaM in buffer B with 1 mM CaCl_2_ and 1 mM ATP and incubated at 30°C for 5 min. After that, to dissociate Ca^2+^/CaM from CaMKII, we added 2 mM EGTA and incubated at 30°C for an additional 5 min. Then, HS-AFM observations were performed in buffer A with 2 mM EGTA and 1 mM ATP. All HS-AFM experiments were performed at room temperature (24° to 26°C).

### HS-AFM image processing and data analysis

HS-AFM images were processed using Fiji (ImageJ) software (National Institutes of Health, USA) (*45*). A 0.5-pixel-radius mean filter was applied to each HS-AFM image to reduce noise. The Template Matching and Slice Alignment plugin for ImageJ was used to correct the drift between images in sequence. The coordinates of the hub assembly center were determined using the Trainable Weka Segmentation plugin (*46*). The MTrackJ (*47*) and TrackMate (*48*) plugins for ImageJ were used to semi-manually track the coordinates of the highest pixel for each kinase domain in all CaMKII protein constructs with all HS-AFM experimental conditions. TrackMate was also used to analyze the number of detectable kinase domains. Because the diameter of the kinase domain of CaMKII is approximately 4 to 5 nm, we surmised that the protrusions with a diameter of approximately 4 nm around the central hub assembly consisted of the kinase domain. Using the TrackMate algorithm (Laplacian of Gaussian detector) (*48*), the number of kinase domains in each frame of the HS-AFM videos was counted, averaged over approximately 150 frames of HS-AFM videos for each condition, and plotted. The distinction between “monomer” and “oligomer” was ascertained by the following criteria. Monomer refers to instances where the overlapping time of kinase domains is limited to two to five frames in AFM images. Conversely, “dimer or oligomer” denotes the continuous overlapping of kinase domains, which persists from the beginning of HS-AFM observations until the final frame during HS-AFM scanning (i.e., ~30 s).

The motions of each kinase domain of rat CaMKIIα (18,924 points in 1577 frames of 144 kinase domains in 12 oligomers), rat nlCaMKIIα (13,740 points in 1145 frames of 96 kinase domains in 8 oligomers), rat nlCaMKIIα_I321E_ (22,632 points in 1886 frames of 156 kinase domains in 13 oligomers), rat CaMKIIα + bosutinib (11,940 points in 995 frames of 84 kinase domains in 7 oligomers), rat CaMKIIα + ADP (12,384 points in 1032 frames of 84 kinase domains in 7 oligomers), rat CaMKIIα + ATP (14,232 points in 1186 frames of 96 kinase domains in 8 oligomers), rat CaMKIIα in EGTA/ADP after Ca^2+^/CaM/ADP stimulation (13,692 points in 1141 frames of 96 kinase domains in 8 oligomers), rat CaMKIIα_T286A_ (18,000 points in 1500 frames of 120 kinase domains in 10 oligomers), rat CaMKIIα_T286A_ in EGTA/ATP after Ca^2+^/CaM/ATP stimulation (17,976 points in 1498 frames of 120 kinase domains in 10 oligomers), rat CaMKIIα_T305A/T306V_ (18,000 points in 1500 frames of 120 kinase domains in 10 oligomers), rat CaMKIIα_T305A/T306V_ in EGTA/ATP after Ca^2+^/CaM/ATP stimulation (18,000 points in 1500 frames of 120 kinase domains in 10 oligomers), hydra CaMKIIα (17,592 points in 1466 frames of 113 kinase domains in 10 oligomers), hydra CaMKIIα in EGTA/ATP after Ca^2+^/CaM/ATP stimulation (17,100 points in 1500 frames of 114 kinase domains in 10 oligomers), *C. elegans* CaMKII (17,148 points in 1429 frames of 120 kinase domains in 10 oligomers), and *C. elegans* CaMKII in EGTA/ATP after Ca^2+^/CaM/ATP stimulation (13,746 points in 1183 frames of 93 kinase domains in 9 oligomers) were analyzed. The motion of Ca^2+^/CaM binding kinase domains in rat CaMKIIα without ATP (4557 points of 1199 frames of 41 kinase domains in 9 oligomers), rat CaMKIIα + ATP (8757 points of 1604 frames of 71 kinase domains in 11 oligomers), rat CaMKIIα_T286A_ + ATP (14,234 points in 1474 frames of 100 kinase domains in 10 oligomers), rat CaMKIIα_T305A/T306V_ + ATP (14,214 points in 1484 frames of 98 kinase domains in 10 oligomers), hydra CaMKIIα +ATP (12,805 points in 1500 frames of 87 kinase domains in 10 oligomers), and *C. elegans* CaMKII + ATP (8,999 points in 1038 frames of 62 kinase domains in 7 oligomers) were analyzed. HS-AFM experiments were repeated at least three times independently with similar results.

### Analysis of kinase domain trajectories

To quantify the trajectory of a single kinase domain, the distance from the center of the hub assembly to the kinase domains (*D*_h-k_) was computed as follows

$$D_{h-k}={\vec{R}}_{k}(n)-{\vec{R}}_{h\mathrm{ub}}(n)$$

where ${\vec{R}}_{k}(n)$ is the kinase domain position in video frame *n* and ${\vec{R}}_{\mathrm{hub}}(n)$ is the center of the hub assembly position in video frame *n*.

To characterize a single kinase domain, the gyration radius, *R_g_*, of the kinase domain trajectory was computed as the root mean square displacement of the kinase domain position from its average position

$$\langle \vec{R}\rangle ,\;i.e.,\;R_{g}^{2}=(1/N)\sum_{n=1}^{N}{\left({\vec{R}}_{k}(n)-\langle \vec{R}\rangle \right)}^{2}$$

where $\left\langle \vec{R}\right\rangle$ is the average position of a single kinase domain.

### Principal components analysis

To visualize the coupled motions of the kinase domains, PCA was performed on their trajectories. Vectors on the kinase domains show the coupled motions along the vectors, and the lengths of the vectors indicate the degree of coupling. PCA was applied using the conventional method (*49*), that is, by diagonalizing the covariance matrix (*C*), defined as follows

$$C_{ij}=\langle ({\vec{r}}_{i}-\langle {\vec{r}}_{i}\rangle )({\vec{r}}_{j}-\langle {\vec{r}}_{j}\rangle )\rangle$$

where $\vec{r}$ represents the *x* and *y* positions of the highest pixel for an individual kinase domain and angular brackets represent the time average. By diagonalizing *C*, eigenvectors were computed.

### Quantification and statistical analysis

All statistical analyses were performed using Igor Pro 9 software (WaveMetrics, USA), OriginPro 2023 (OriginLab, USA), or GraphPad Prism (GraphPad Software Inc., USA). A significance level of α = 0.05 was used for all analyses, and *P* values were adjusted for multiple comparisons where relevant. Unless otherwise noted, the normality of distributions was tested by the Shapiro-Wilk test. When it failed, the Kruskal-Wallis test and Mann-Whitney’s *U* test were used to compare the two groups. When several conditions were compared, one-way analysis of variance (ANOVA) and the Kruskal-Wallis test were used for the analysis of multiple groups with a single independent variable. The Dunnett’s and Dunn’s tests were used as follow-up tests to the Kruskal-Wallis test, where Dunn’s test was used to compare every mean with every other mean, and the Dunnett’s test was used to compare every mean to a control mean. The *F* test was used to compare the variance of two groups: *P* > 0.05, not significant; *P < 0.05, **P < 0.01, and ***P < 0.001 were considered statistically significant. Data are represented as the means ± SDs.

## Notes

View/request a protocol for this paper from *Bio-protocol*.
