# Supplementary material for: Imaging single CaMKII holoenzymes at work by high-speed atomic force microscopy
Source: Sci Adv. 2023 Jun 30;9(26):eadh1069. doi: 10.1126/sciadv.adh1069 (PMC10313165; doi:10.1126/sciadv.adh1069)
Supplement: Supplementary file 1 — Figs. S1 to S13 Legends for movies S1 to S7 [file sciadv.adh1069_sm.pdf]

Science Advances
AAAS

Supplementary Materials for

Imaging single CaMKII holoenzymes at work by high-speed atomic force microscopy

Shotaro Tsujioka *et al.*

Corresponding author: Hideji Murakoshi, murakosh@nips.ac.jp;
Mikihiro Shibata, msshibata@staff.kanazawa-u.ac.jp

Sci. Adv. **9**, eadh1069 (2023)
DOI: 10.1126/sciadv.adh1069

The PDF file includes:

Figs. S1 to S13
Legends for movies S1 to S7

Other Supplementary Material for this manuscript includes the following:

Movies S1 to S7

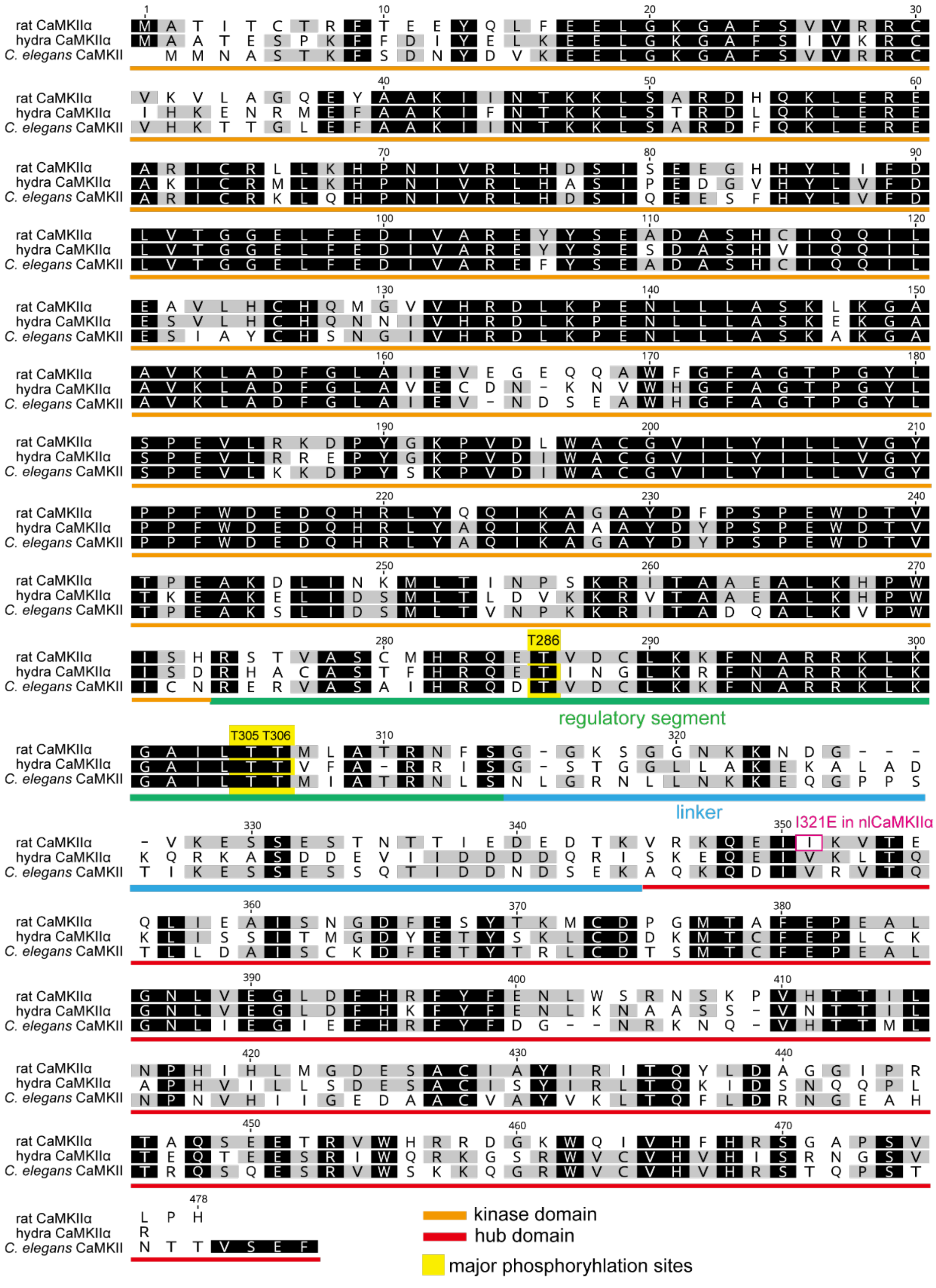

Fig. S1. Sequence alignment of CaMKII species used in this study.

Three CaMKII species are shown: rat CaMKIIα (*Rattus norvegicus*, Accession#: NP_037052.1), hydra CaMKIIα isoform X2 (*Hydra vulgaris*, Accession#: XP_012553992.1), and *C. elegans* CaMKII (*Caenorhabditis elegans*, Accession#: NP_001379280.1).

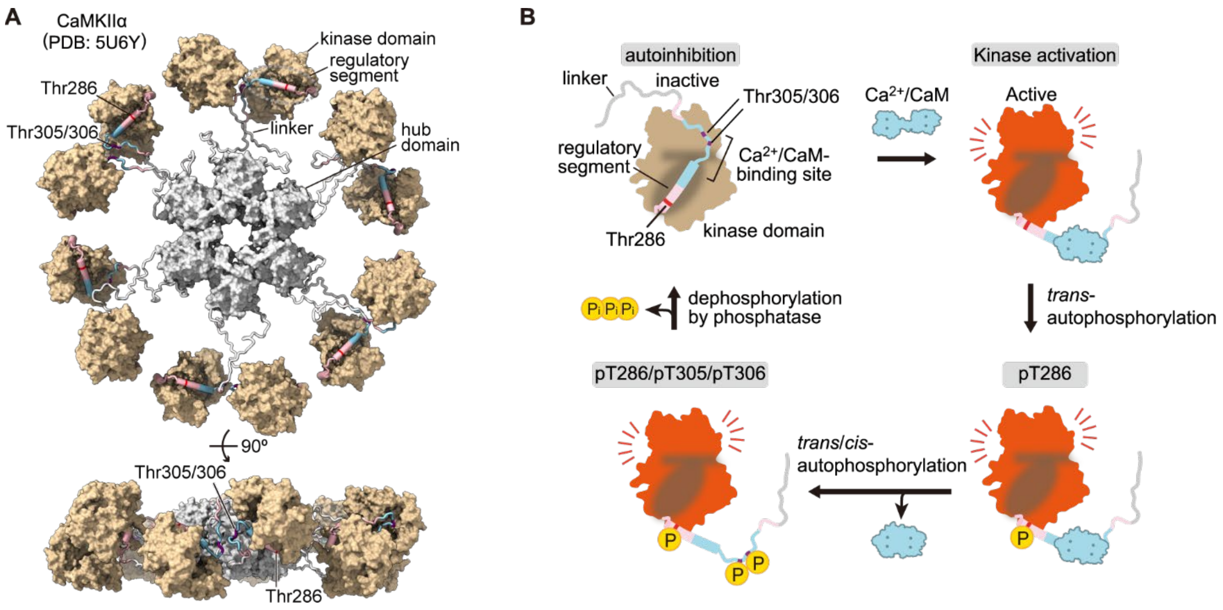

Fig. S2. Illustration of CaMKIIα architecture.

(**A**) Pseudoatomic model of CaMKIIα dodecamer from EM data (*11*). The solvent-excluded surface is represented. The hub domains (light gray surface, residues 345–472), kinase domains (tan surface, residues 1–273), regulatory segment (magenta cylinder, residues 274–314) with Ca^{2+}/CaM-binding site (blue cylinder, residues 293–310) and linker region (white ribbon, residues 315–344) are shown. Phosphorylation sites at Thr286, Thr305, and Thr306 are shown in red and purple.

(**B**) Illustration of the state change of CaMKIIα (only a single kinase domain is shown). In an inactive state, the kinase domain is autoinhibited by the binding of the regulatory segment (autoinhibition). Ca^{2+}/CaM binding leads to the release of the regulatory segment and the appearance of the substrate-binding site (dark tan) (i.e., kinase activation). The concomitant activation of adjacent kinases leads to autophosphorylation at Thr286 (autophosphorylation at pT286). After Ca^{2+}/CaM dissociation, autophosphorylation at Thr305/306 occurs. Finally, a protein phosphatase dephosphorylates CaMKIIα and returns to the inactive state.

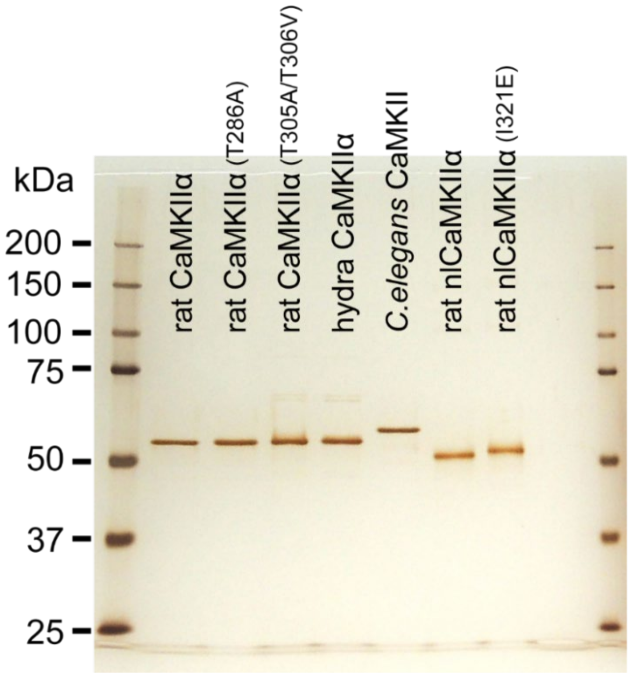

Fig. S3. The purity of the CaMKII proteins used in this study was confirmed by silver staining.

CaMKII holoenzymes in the absence of ADP/ATP were purified from HEK-293 cells with two-step purification using His and Strep tags.

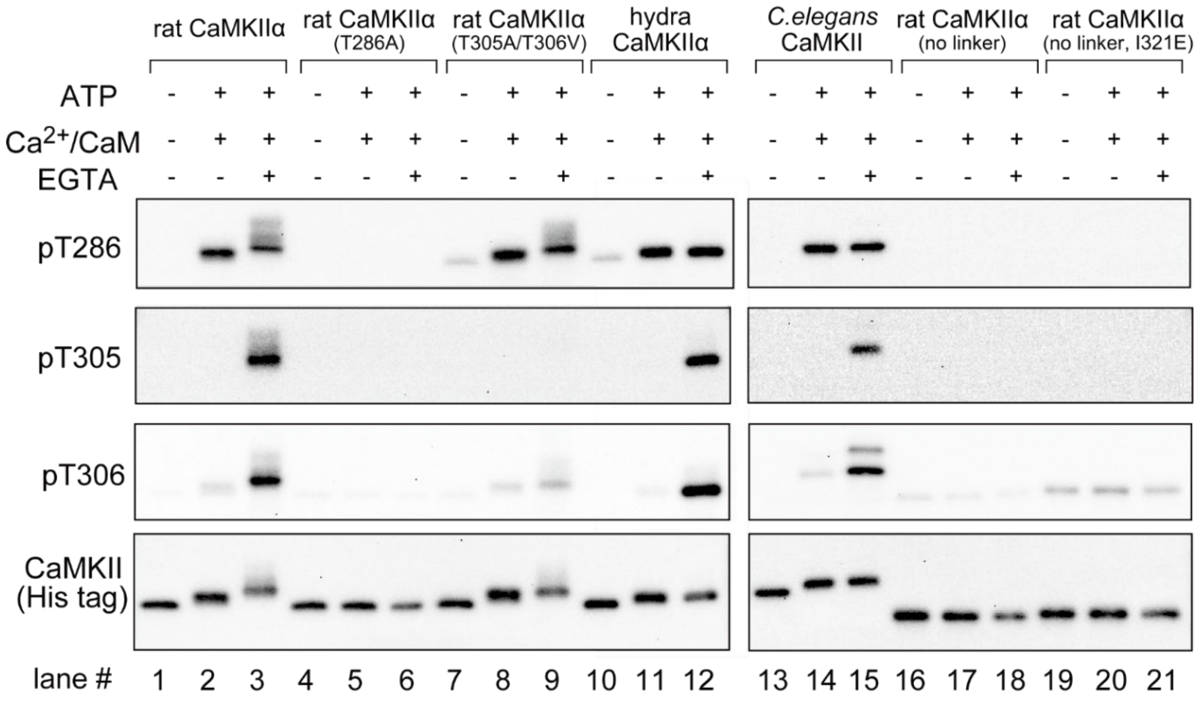

Fig. S4. Western blotting of purified CaMKII phosphorylation.

(lanes #1, 4, 7, 10, 13, 16, 19) Purified proteins were loaded without activation.

(lanes #2, 5, 8, 11, 14, 17, 20) CaMKII proteins (50 nM) were incubated with 800 nM CaM, 1 mM $CaCl_2$, and 1 mM ATP in a reaction buffer (50 mM Tris-HCl, pH 7.4, 150 mM KCl, and 10 mM $MgCl_2$) at 30°C for 5 min. This protocol selectively induces phosphorylation at Thr286 but not Thr305/306.

(lanes #3, 6, 9, 12, 15, 18, 21) Ca^{2+}/CaM was dissociated by incubation with 2 mM EGTA for 5 min at 30°C and an additional 10 min at 25°C. This protocol induces phosphorylation at Thr305/306 and kinase domain oligomerization for rat CaMKIIα (lane #3).

The amount of protein loaded was assessed with an anti-His tag antibody.

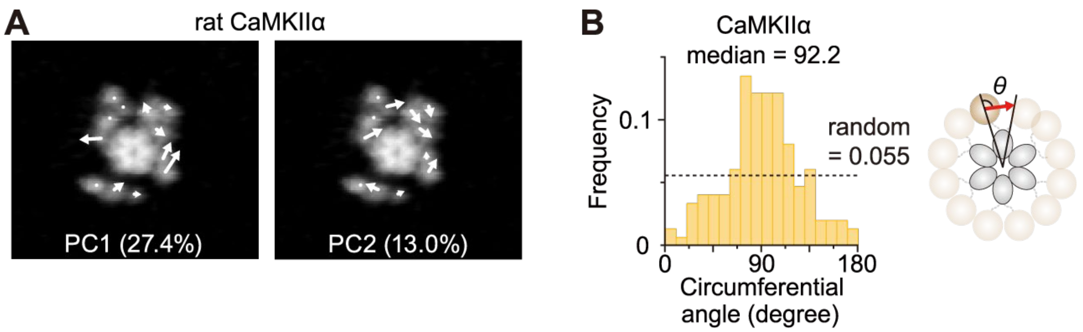

Fig. S5. Kinase domains of the rat CaMKIIα oligomer exhibit circumferential motion around the hub assembly.

(**A**) PCA of kinase domain motion in rat CaMKIIα. The two largest principal components of kinase domain movement are shown (PC1 and PC2). Arrows illustrate eigenvectors indicating the direction and magnitude of the collective motions of kinase domains determined by HS-AFM.

(**B**) Direction of kinase domain motions in rat CaMKIIα oligomers determined from PCA. Black dotted lines represent the random distribution.

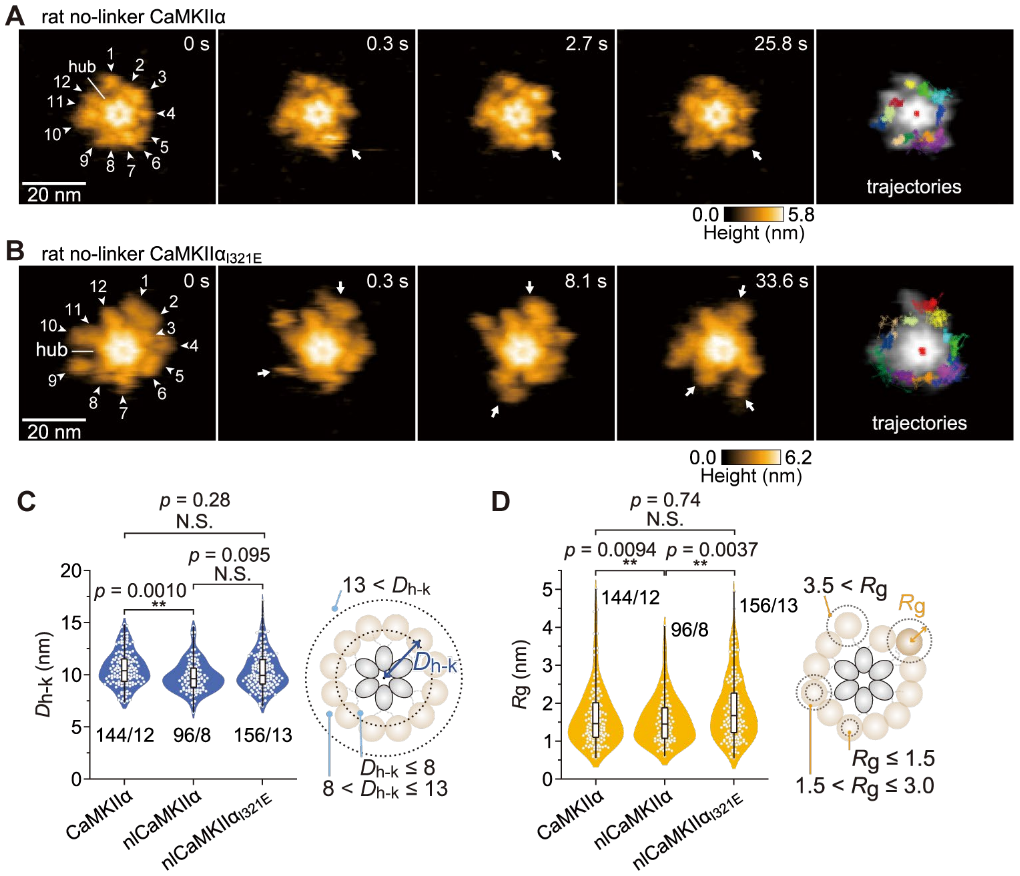

Fig. S6. Kinase domain motion of the rat nlCaMKIIα and nlCaMKIIα$_{I321E}$ mutants.

(**A** and **B**) Sequential HS-AFM images of rat no-linker CaMKIIα (nlCaMKIIα) (A; see also movie S1B) and rat no-linker CaMKIIα$_{I321E}$ (nlCaMKIIα$_{I321E}$) (B; see also movie S1C). White arrowheads with arbitrary numbers 1 to 12 indicate kinase domains in the oligomers. White arrows indicate the motions of kinase domains in the oligomers. Frame rate, 3.3 frames/s. Trajectories of the center of the hub assembly (red in the center) and the kinase domains in rat nlCaMKIIα (A) and rat nlCaMKIIα$_{I321E}$ (B) were tracked for ~30 s (right). Trajectories that remained roughly circular represent kinase domains that moved in a narrow range, while trajectories that covered a larger area correspond to single kinase domains that moved in a wide range.

(**C** and **D**) Distances from the center of the hub assembly to kinase domains ($D_{h\text{-}k}$) (C) and R_g (D) in rat CaMKIIα, rat nlCaMKIIα, and rat nlCaMKIIα$_{I321E}$. The number of samples (kinases/holoenzymes) is indicated in the figure. N.S., not significant. $^{**}p < 0.01$ (Kruskal–Wallis test with Dunn's post hoc test) (C). $^{**}p < 0.01$ (F-test) (D). HS-AFM experiments were repeated at least three times independently with similar results.

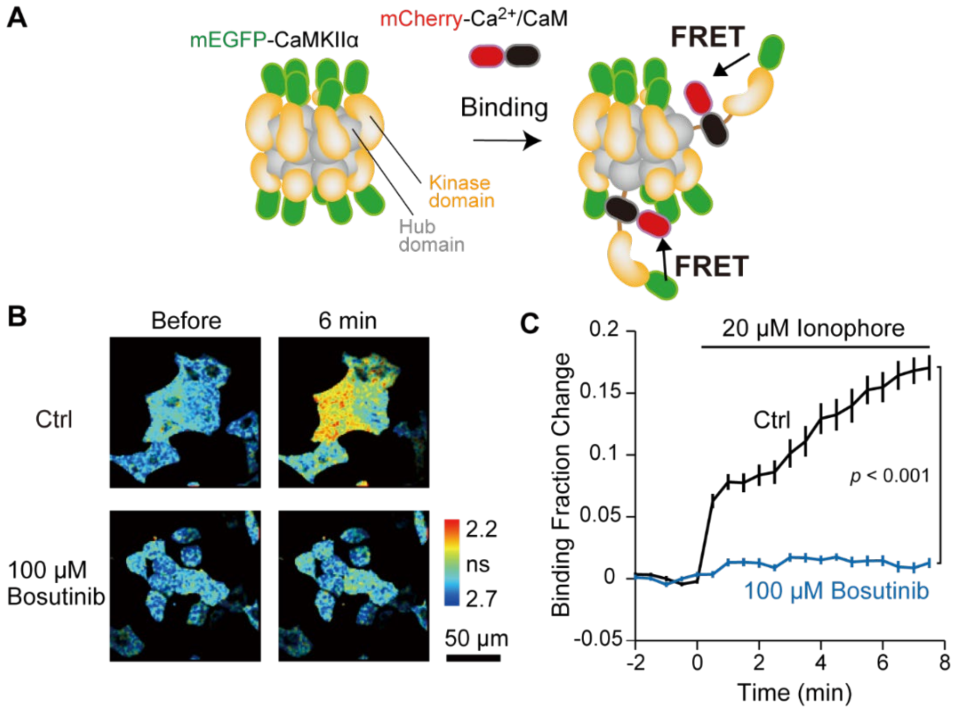

Fig. S7. Bosutinib inhibits the binding between CaMKIIα and CaM in HeLa cells.

(**A**) Schematic drawing of the experimental design. FRET between mEGFP-fused CaMKIIα (mEGFP-CaMKIIα) and mCherry-fused CaM was monitored by 2-photon fluorescence lifetime microscopy.

(**B**) Representative fluorescence lifetime images of HeLa cells expressing mEGFP-CaMKIIα and mCherry-CaM. The FRET donor and acceptor plasmids were transfected at a ratio of 1:3. To induce binding between mEGFP-CaMKIIα and mCherry-CaM, 20 μM ionophore was bath applied in the absence or presence of 100 μM bosutinib. Bosutinib was incubated for 40–50 min before the experiments and during the observation. The warmer color indicates the binding between mEGFP-CaMKIIα and mCherry-CaM.

(**C**) Time course of the binding fraction change after ionophore application. The number of cells analyzed was 28 for Ctrl and 33 for bosutinib. The data are shown as the mean ± sem with a statistical comparison at 7.5 min (*t* test).

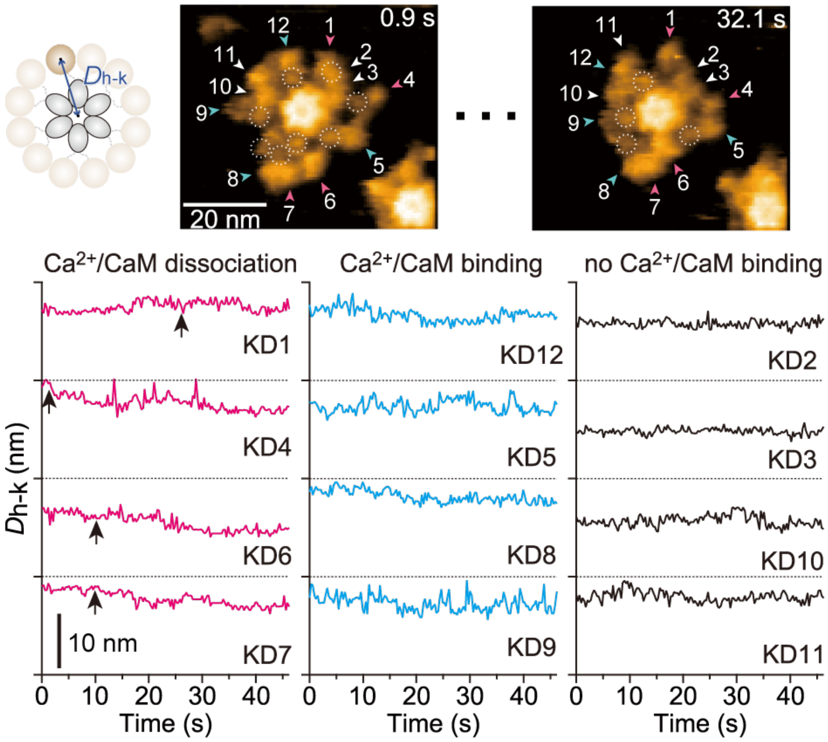

Fig. S8. Ca^{2+}/CaM binding kinase domains form the extended structure in the rat CaMKIIα oligomer.

Time course of $D_{h\text{-}k}$ of kinase domains in rat CaMKIIα (50 nM) with Ca^{2+}/CaM (1 mM Ca^{2+}, 800 nM CaM). Arbitrary numbers from 1 to 12 indicate kinase domains. White, blue, and magenta arrowheads indicate kinase domains with no binding, binding, and dissociation of Ca^{2+}/CaM, respectively. Black arrows in the time course indicate Ca^{2+}/CaM dissociation from rat CaMKIIα. KD, kinase domain. HS-AFM experiments were repeated at least three times independently with similar results.

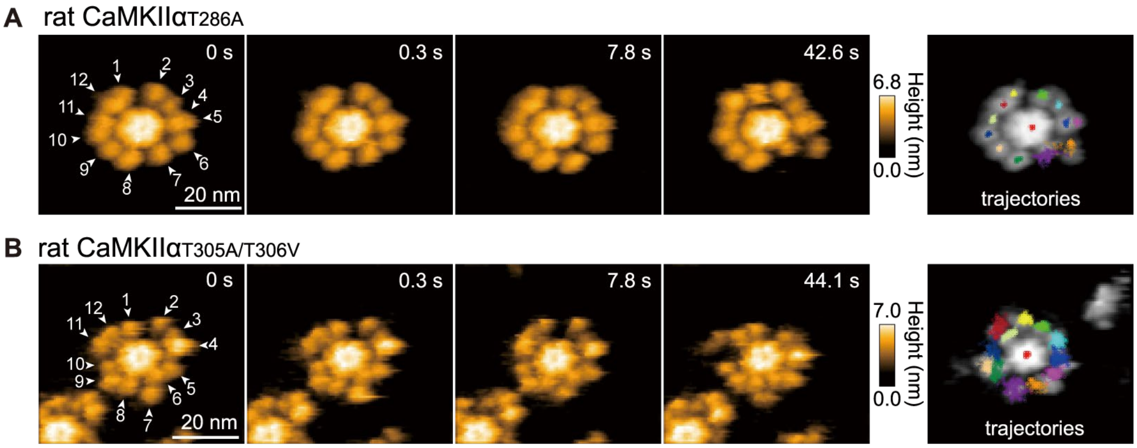

Fig. S9. Kinase domain motion of the rat CaMKIIα$_{T286A}$ and CaMKIIα$_{T305A/T306V}$ mutants.

(**A** and **B**) Sequential HS-AFM images of the T286A (A) and T305A/T306V (B) rat CaMKIIα mutants. White arrowheads with arbitrary numbers 1 to 12 indicate kinase domains in the oligomers. Frame rate, 3.3 frames/s. Trajectories of the center of the hub assembly (red in the center) and the kinase domains were tracked for ~30 s (right).

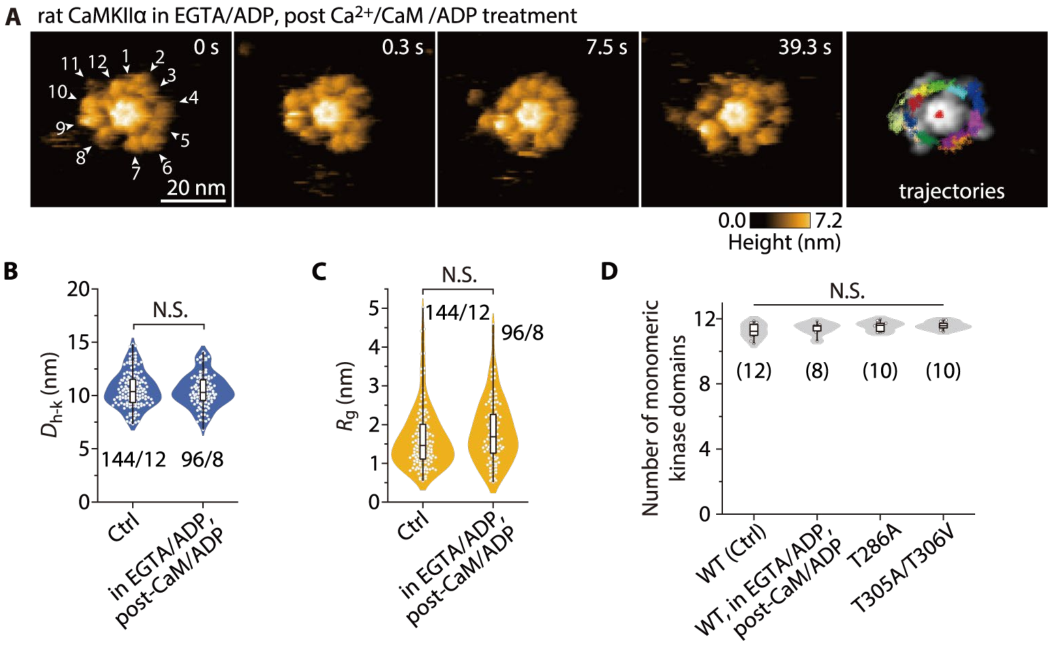

Fig. S10. Kinase domain motion of rat CaMKIIα after Ca^{2+}/CaM and ADP stimulation.

(**A**) Sequential HS-AFM images of rat CaMKIIα in EGTA (2 mM) after Ca^{2+}/CaM (1 mM Ca^{2+}, 800 nM CaM) and ADP (1 mM) stimulation. White arrowheads with arbitrary numbers from 1 to 12 indicate kinase domains in the oligomers. Frame rate, 3.3 frames/s. Trajectories of the center of the hub assembly (red in the center) and the kinase domains in rat CaMKIIα in EGTA post-Ca^{2+}/CaM and ADP stimulation were tracked for ~30 s (right).

(**B** and **C**) Distances from the center of the hub assembly to kinase domains $D_{h\text{-}k}$ (B) and gyration radius R_g (C) for rat CaMKIIα and rat CaMKIIα in EGTA after Ca^{2+}/CaM and ADP incubation. The number of samples (kinases/holoenzymes) is indicated in the figure. N.S., not significant (Kruskal–Wallis test).

(**D**) Number of detectable kinase domains as the 4 nm object surrounding the hub assembly in rat CaMKIIα (WT, Ctrl), WT in EGTA + ADP post Ca^{2+}/CaM+ADP, CaMKIIα$_{T286A}$, and CaMKIIα$_{T305/306A}$. The number of samples (holoenzymes) is indicated in the figure. No significant difference was observed (one-way ANOVA).

HS-AFM experiments were repeated at least three times independently with similar results.

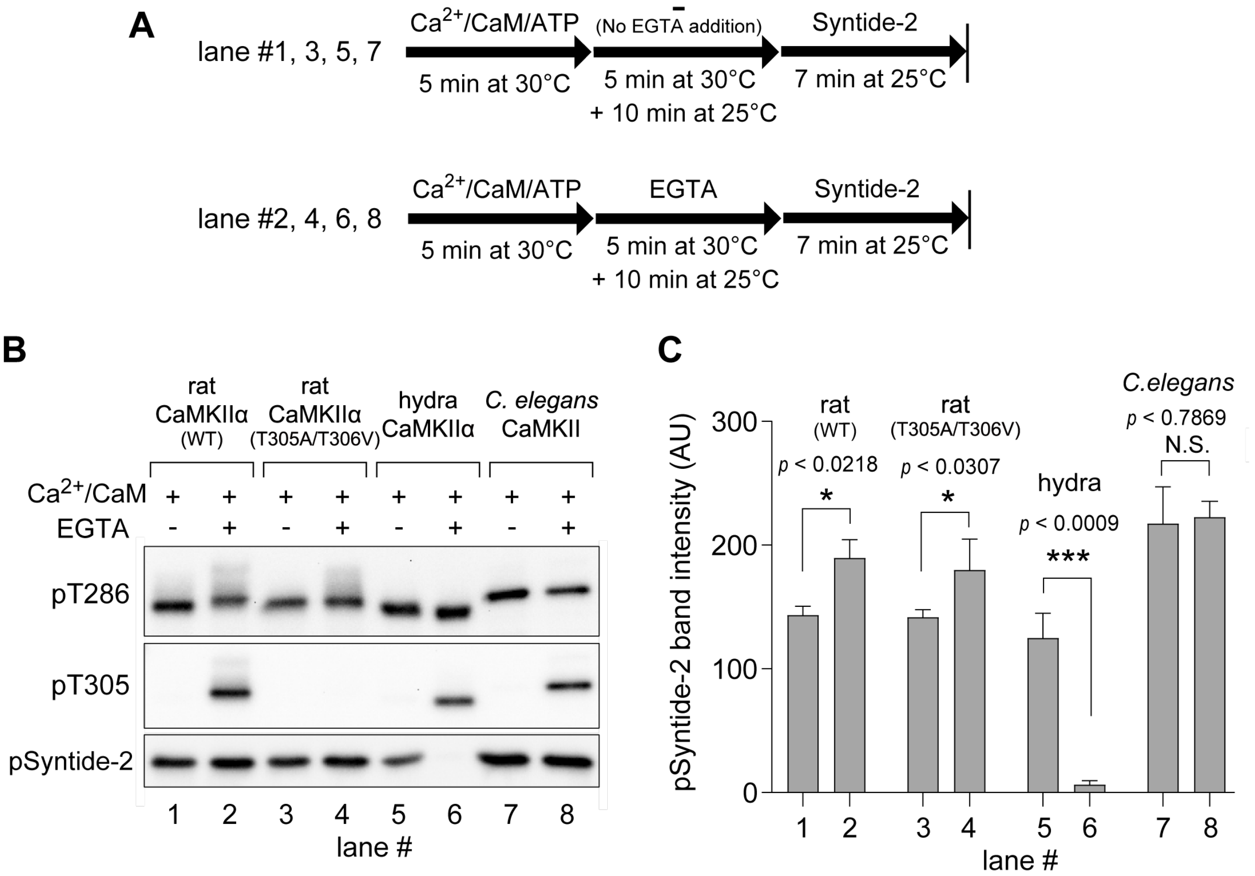

Fig. S11. Kinase assay of pT286/pT305/pT306 CaMKII holoenzymes.

(**A** and **B**) Phosphorylation of the sfGFP-syntide-2 peptide by pT286/pT305/pT306 CaMKII species was detected by western blotting. pT286 CaMKII (lanes 1, 3, 5, 7) and pT286/pT305/pT306 CaMKII (lanes 2, 4, 6, 8) were prepared as described in the Materials and Methods. This protocol also induces kinase domain oligomerization in rat CaMKIIα holoenzymes (Fig. 3B). Subsequently, 1 μM sfGFP-Syntide-2 was incubated for 7 min at 25°C as a substrate for CaMKII.

(**C**) Quantification of the experiments. For data analysis, kinase activity. Error bars indicate SEM for four independent experiments. N.S. (not significant, $p > 0.05$); *$p < 0.05$; ***$p < 0.001$; paired *t* test.

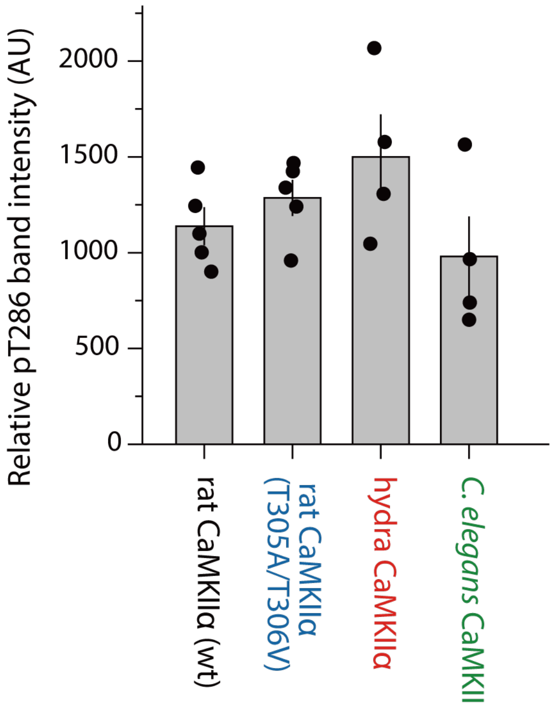

Fig. S12. Statistical comparison of the pT286 band intensities (0 unit/µL PP2A) of pT286/pT305/pT306 CaMKII holoenzymes.

The data set used in Fig. 5C to D was also employed here. Error bars indicate SEM. No significant difference was observed (one-way ANOVA).

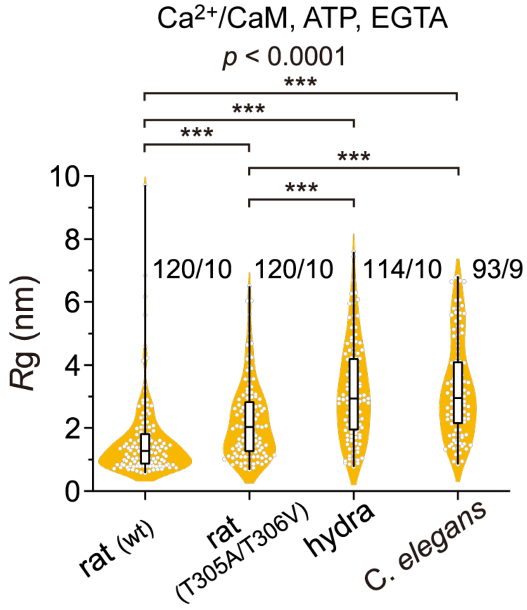

Fig. S13. Statistical comparison of R_g of the fully phosphorylated CaMKII from different species.

The data set used in Fig. 3G and Fig. 4H was also employed here. $^{*}p < 0.05$, $^{***}p < 0.001$ (Kruskal–Wallis test with Dunn's post hoc test). The number of samples (kinases/holoenzymes) is indicated in the figure.

Movie S1. HS-AFM videos of three representative rat CaMKIIα$_{WT}$ and no-linker CaMKIIα molecules on a P[5]A+-modified mica surface. (A) Rat CaMKIIα$_{WT}$ (50 nM). (**B**) Rat no-linker CaMKIIα (nlCaMKIIα). (**C**) Rat no-linker CaMKIIα$_{I321E}$ (nlCaMKIIα$_{I321E}$). The trajectories of the kinase domains in the rat CaMKIIα oligomers are also shown at the bottom. Image size, 110 × 98 pixels^2; scan area, 62 × 55 nm^2; frame rate, 3.3 fps.

Movie S2. HS-AFM videos of three representative rat CaMKIIα$_{WT}$ molecules on a P[5]A+-modified mica surface. (**A**) Rat CaMKIIα$_{WT}$ (50 nM) without any addition of inhibitors or ligands (ctrl). These HS-AFM videos are the same as Movie S1A. (**B, C**) Rat CaMKIIα$_{WT}$ treated with 50 μM bosutinib (B) and Ca^{2+}/CaM (1 mM Ca^{2+}, 800 nM CaM (C). The trajectories of the kinase domains in the CaMKIIα oligomers are also shown at the bottom. Image size, 110 × 98 pixels^2; scan area, 62 × 55 nm^2; frame rate, 3.3 fps.

Movie S3. HS-AFM videos of three representative rat CaMKIIα$_{WT}$ molecules after phosphorylation on a P[5]A+-modified mica surface. (**A**) Rat CaMKIIα$_{WT}$ (50 nM) (ctrl). These HS-AFM videos are the same as MovieS1A. (**B, C**) Rat CaMKIIα$_{WT}$ treated with Ca^{2+}/CaM + ATP (1 mM Ca^{2+}, 800 nM CaM, 1 mM ATP) (B) and EGTA (2 mM) + ATP (1 mM) after Ca^{2+}/CaM/ATP stimulation (1 mM Ca^{2+}, 800 nM CaM, 1 mM ATP) (C). The trajectories of the kinase domains in the CaMKIIα oligomers are also shown at the bottom. Dotted white circles indicate Ca^{2+}/CaMs bound to CaMKIIα during the first 9.9 s. Image size, 110 × 98 pixels^2; scan area, 62 × 55 nm^2; frame rate, 3.3 fps.

Movie S4. HS-AFM videos of three representative rat CaMKIIα$_{T286A}$ molecules on a P[5]A+-modified mica surface. (**A**) Rat CaMKIIα$_{T286A}$ (50 nM) (ctrl). (**B, C**) Rat CaMKIIα$_{T286A}$ treated with Ca^{2+}/CaM + ATP (1 mM Ca^{2+}, 800 nM CaM, 1 mM ATP) (B) and EGTA (2 mM) + ATP (1 mM) after Ca^{2+}/CaM/ATP stimulation (1 mM Ca^{2+}, 800 nM CaM, 1 mM ATP) (C). The trajectories of the kinase domains in the CaMKIIα oligomers are also shown at the bottom. Dotted white circles indicate Ca^{2+}/CaMs bound to CaMKIIα during the first 9.9 s. Image size, 110 × 98 pixels^2; scan area, 62 × 55 nm^2; frame rate, 3.3 fps.

Movie S5. HS-AFM videos of three representative rat CaMKIIα$_{T305A/T306V}$ molecules on a P[5]A+-modified mica surface. (**A**) Rat CaMKIIα$_{T305A/T306V}$ (50 nM) (ctrl). (**B, C**) Rat CaMKIIα$_{T305A/T306V}$ treated with Ca^{2+}/CaM + ATP (1 mM Ca^{2+}, 800 nM CaM, 1 mM ATP) (B) and in EGTA (2 mM) + ATP (1 mM) after Ca^{2+}/CaM/ATP stimulation (1 mM Ca^{2+}, 800 nM CaM, 1 mM ATP) (C). The trajectories of the kinase domains in the CaMKIIα oligomers are also shown at the bottom. Dotted white circles indicate Ca^{2+}/CaMs bound to CaMKIIα during the first 9.9 s. Image size, 110 × 98 pixels^2; scan area, 62 × 55 nm^2; frame rate, 3.3 fps.

Movie S6. HS-AFM videos of three representative hydra CaMKIIα$_{WT}$ molecules on a P[5]A+-modified mica surface. (**A**) hydra CaMKIIα$_{WT}$ (50 nM) (ctrl). (**B, C**) Hydra CaMKIIα$_{WT}$

treated with Ca^{2+}/CaM + ATP (1 mM Ca^{2+}, 800 nM CaM, 1 mM ATP) (B) and EGTA (2 mM) + ATP (1 mM) after Ca^{2+}/CaM/ATP stimulation (1 mM Ca^{2+}, 800 nM CaM, 1 mM ATP) (C). The trajectories of the kinase domains in the CaMKIIα oligomers are also shown at the bottom. Dotted white circles indicate Ca^{2+}/CaMs bound to CaMKIIα during the first 9.9 s. Image size, 110 × 98 pixels^2; scan area, 62 × 55 nm^2; frame rate, 3.3 fps.

Movie S7. HS-AFM videos of three representative *C. elegans* CaMKII$_{WT}$ molecules on a P[5]A+-modified mica surface. (**A**) *C. elegans* CaMKII$_{WT}$ (50 nM) (ctrl). (**B, C**) *C. elegans* CaMKII$_{WT}$ treated with Ca^{2+}/CaM + ATP (1 mM Ca^{2+}, 800 nM CaM, 1 mM ATP) (B) and EGTA (2 mM) + ATP (1 mM) after Ca^{2+}/CaM/ATP stimulation (1 mM Ca^{2+}, 800 nM CaM, 1 mM ATP) (C). The trajectories of the kinase domains in the CaMKIIα oligomers are also shown at the bottom. Dotted white circles indicate Ca^{2+}/CaMs bound to CaMKIIα during the first 9.9 s. Image size, 110 × 98 pixels^2; scan area, 62 × 55 nm^2; frame rate, 3.3 fps.
